# Layered Pd oxide on PdSn nanowires for boosting direct H_2_O_2_ synthesis

**DOI:** 10.1038/s41467-022-33757-0

**Published:** 2022-10-14

**Authors:** Hong-chao Li, Qiang Wan, Congcong Du, Jiafei Zhao, Fumin Li, Ying Zhang, Yanping Zheng, Mingshu Chen, Kelvin H. L. Zhang, Jianyu Huang, Gang Fu, Sen Lin, Xiaoqing Huang, Haifeng Xiong

**Affiliations:** 1 The State Key Laboratory of Physical Chemistry of Solid Surfaces, iChEM (Collaborative Innovation Center of Chemistry for Energy Materials), Department of Chemistry, College of Chemistry & Chemical Engineering, Xiamen University, Xiamen, 361005 China; 2 Innovation Laboratory for Sciences and Technologies of Energy Materials of Fujian Province (IKKEM), 4221 Xiangan South Road, Xiamen, 361102 P. R. China; 3 State Key Laboratory of Photocatalysis on Energy and Environment, College of Chemistry, Fuzhou University, Fuzhou, 350002 China; 4 Clean Nano Energy Center, State Key Laboratory of Metastable Materials Science and Technology, Yanshan University, Qinhuangdao, 066004 China

**Keywords:** Heterogeneous catalysis, Catalyst synthesis, Catalytic mechanisms

## Abstract

Hydrogen peroxide (H_2_O_2_) has the wide range of applications in industry and living life. However, the development of the efficient heterogeneous catalyst in the direct H_2_O_2_ synthesis (DHS) from H_2_ and O_2_ remains a formidable challenge because of the low H_2_O_2_ producibility. Herein, we develop a two-step approach to prepare PdSn nanowire catalysts, which comprises Pd oxide layered on PdSn nanowires (Pd_L_/PdSn-NW). The Pd_L_/PdSn-NW displays superior reactivity in the DHS at zero Celcius, presenting the H_2_O_2_ producibility of 528 mol kg_cat_^−1^·h^−1^ and H_2_O_2_ selectivity of >95%. A layer of Pd oxide on the PdSn nanowire generates bi-coordinated Pd, leading to the different adsorption behaviors of O_2_, H_2_ and H_2_O_2_ on the Pd_L_/PdSn-NW. Furthermore, the weak adsorption of H_2_O_2_ on the Pd_L_/PdSn-NW contributes to the low activation energy and high H_2_O_2_ producibility. This surface engineering approach, depositing metal layer on metal nanowires, provides a new insight in the rational designing of efficient catalyst for DHS.

## Abstract (web summary)

The development of the efficient catalyst in the direct H2O2 synthesis (DHS) from H2 and O2 remains a formidable challenge. Here, the authors develop a two-step approach to prepare a layer of Pd oxide on PdSn nanowires which displays superior reactivity in the DHS at zero Celcius.

## Introduction

Hydrogen peroxide (H_2_O_2_) is widely used as oxidant in many fields because it is environmentally friendly and the byproducts only involve H_2_O and O_2_, as compared to other oxidants (e.g. Cl-containing oxidants and nitric acid)^1,2^. Currently, the anthraquinone oxidation process (AO process) is employed as the method to produce H_2_O_2_ in industry, whereas the AO process requires highly intense energy consumption and the addition of toxic organic solvents, such as alkylbenzene and so on. Therefore, it is desirable to develop sustainable process and more efficient strategy towards the production of H_2_O_2_^3–7^.

Direct H_2_O_2_ synthesis (DHS) from H_2_ and O_2_ is an effective alternative for small-scale and on-site H_2_O_2_ production, which is usually performed using Pd-based catalyst^8–11^. However, the catalytic activity was limited due to the high-side reactions such as hydrogenation and decomposition when employing a pure Pd-based catalyst, leading to a decreased net yield. Numerous efforts have been devoted to developing high-performance catalysts towards DHS, such as the use of bimetallic Pd-Au^12–15^, trimetallic Pd-Au-Pt^16^, acid and halide additives^17–21^. However, these Pd catalysts contain expensive gold and the H_2_O_2_ yield is still far from that achieved by the AO process^22–24^. The addition of Sn to Pd catalysts has attracted intensive attention due to the high stability and inert hydrogenation with Sn component for the H_2_O_2_ synthesis^3^. In particular, the synthesis of PdSn nanocatalyst involved a multi-step protocol of oxidation-reduction-oxidation (O-R-O), and a tin oxide surface layer that encapsulates small Pd-rich particles was formed while leaving larger PdSn alloy particles exposed. The PdSn nanocatalyst prepared via O-R-O presented the H_2_O_2_ production with high selectivity of >95%, while only showing the H_2_O_2_ producibility of ~70 mmol·g_cat_^−1^·h^−1^.

Metal nanowires (NWs) is one-dimensional (1D) structure material, which has been utilized in photonic^25,26^, electrical^27,28^, and plasmonic-related applications^28,29^. As compared to nanoparticles or bulk material, these 1D nanowire structures can expedite orientable electronic or ions transfer and diffusion to promote catalytic kinetics. Therefore, nanowires can provide a unique platform to study catalysis^30,31^. For example, in a nanowire-bacteria hybrid system, nanowires can capture electrons and deliver them to bacteria, allowing bacteria to ensure the conversion of CO_2_^26^. On the other hand, cobalt oxide (CoO) nanorods/nanowires were reported to create oxygen vacancies on the nanofacets^27^. The catalyst exhibits excellent electrocatalytic ORR/OER performance due to the modulated electronic structure of cobalt oxide nanorods/nanowires revealed by simulation.

**Fig. 1:**
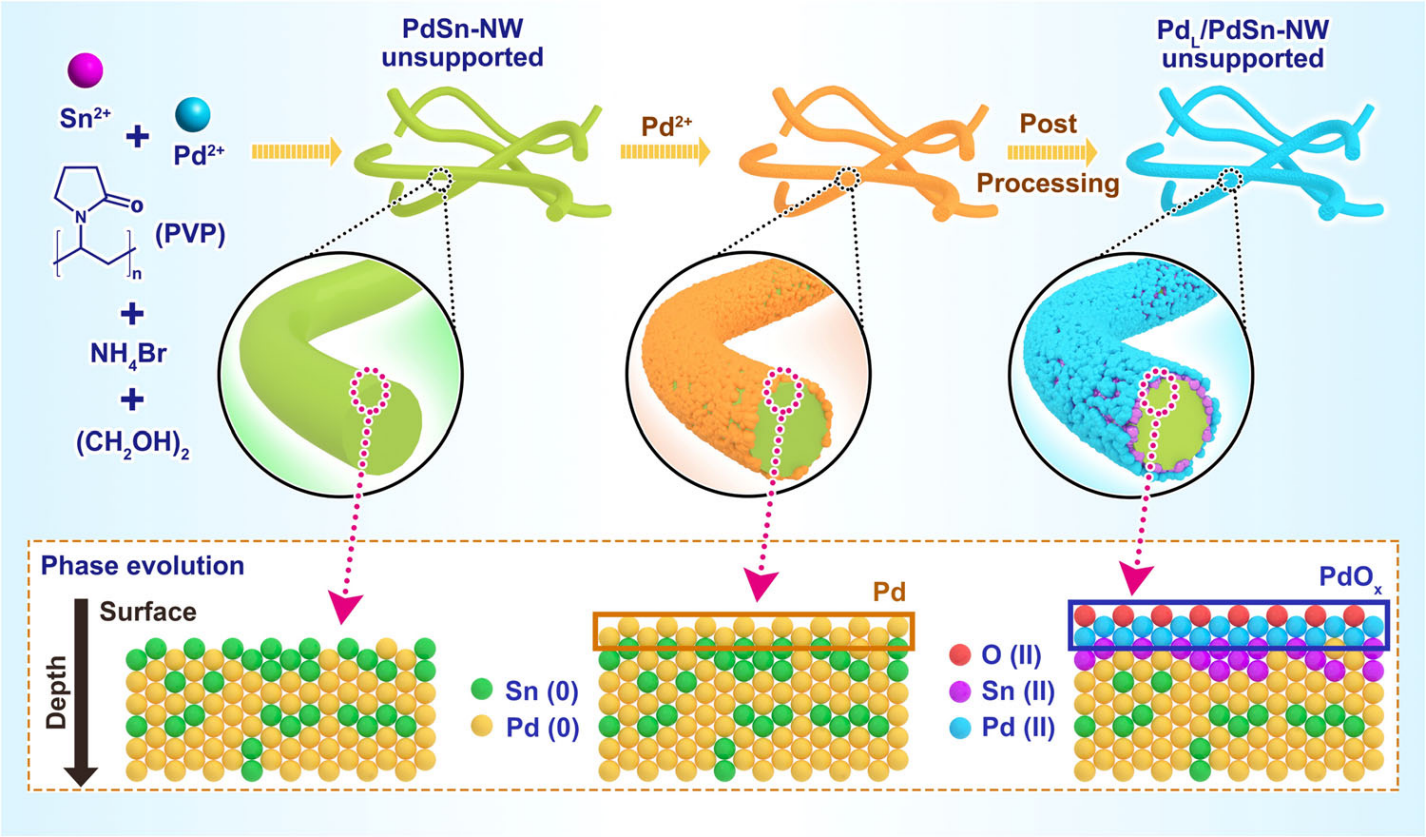
Schematic illustration of the synthesis of the unsupported PdSn-NW and Pd_L_/PdSn-NW. The supported PdSn-NW and Pd_L_/PdSn-NW catalysts are supported on TiO_2_. The two-step protocol involving an annealing process was used for the synthesis of Pd_L_/PdSn-NW. PdSn nanowire (NW) was prepared with a Pd: Sn molar ratio of 4 firstly and then, Pd precursor was deposited on the as-prepared PdSn nanowires again, followed by depositing on TiO_2_ and a rapid annealing in air. A PdSn-NW catalyst with Pd:Sn ratio of 4 was also synthesized for the purpose of comparison via one-step method by mixing Sn^2+^, Pd^2+^, PVP, EG, and NH_4_Br.

**Fig. 2:**
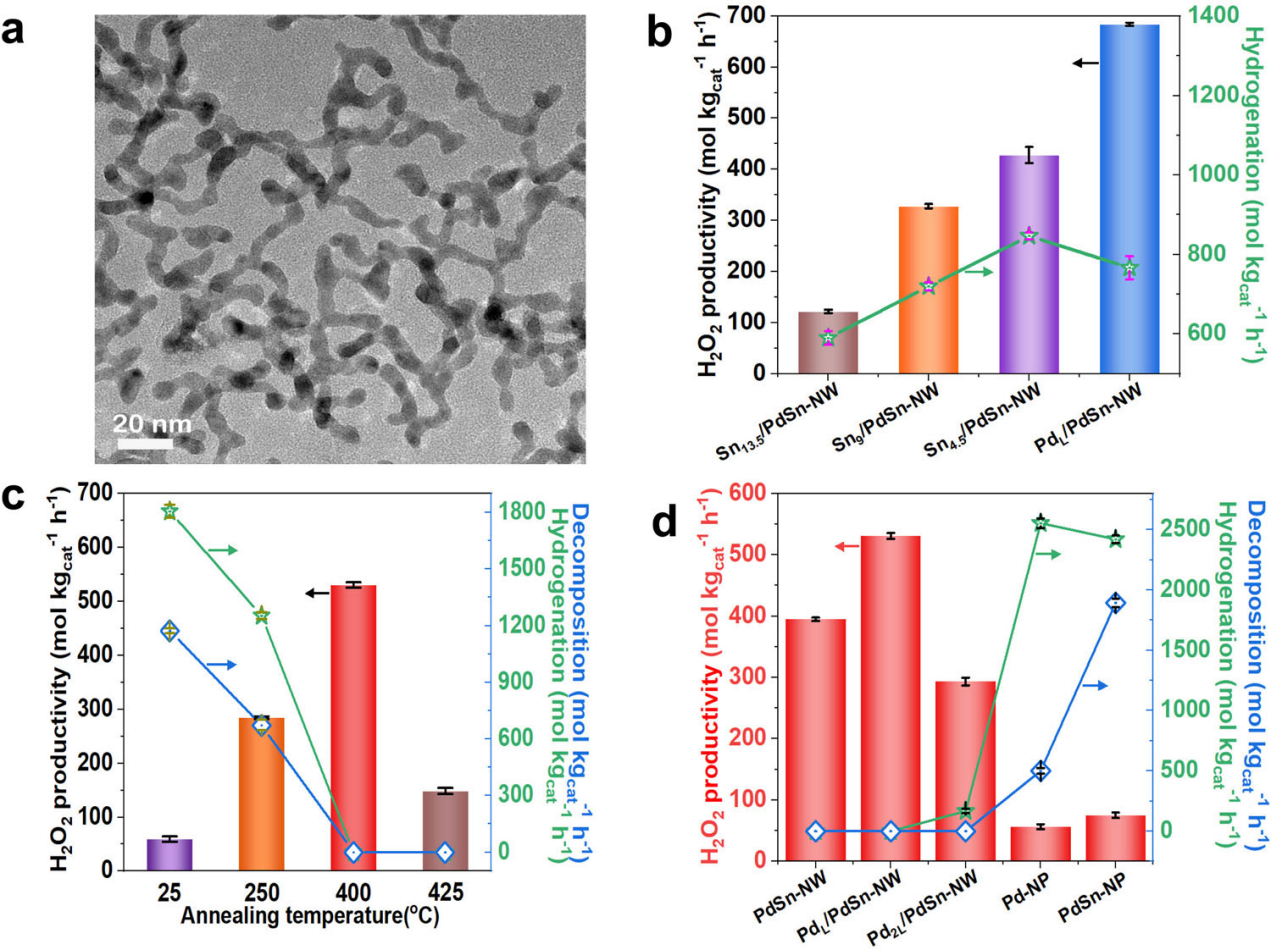
Representative TEM image and the catalytic performances of the PdSn nanowire catalysts in the direct H_2_O_2_ synthesis (DHS). **a** Representative TEM image of the unsupported PdSn nanowire catalyst prepared by two-step (unsupported Pd_L_/PdSn-NW). **b** H_2_O_2_ producibility of the supported Pd_L_/PdSn-NW and Sn_x_/PdSn-NW catalysts with different Pd/Sn ratios after annealing in air (350 °C, 8 min), demonstrating the addition of Sn has negative effect on the producibility of H_2_O_2_. **c** H_2_O_2_ producibility, hydrogenation, and decomposition of the supported Pd_L_/PdSn-NW catalyst annealing at different temperatures in air, showing that the supported Pd_L_/PdSn-NW annealing at 400 °C did not catalyze the hydrogenation and decomposition. **d** The comparison of the H_2_O_2_ producibility, hydrogenation, and decomposition of supported Pd_L_/PdSn-NW catalyst with other Pd catalysts (Table 1). The error bars in **b**–**d** show the standard deviation in the measurements. The standard deviation was achieved from the repeated runs of three to five times using fresh catalyst each time. The error bars refers to the standard deviation of multiple times measurements of hydrogen peroxide productivity.

Herein, we develop an approach to prepare PdSn nanowires to directly produce H_2_O_2_ and found that a layer of Pd oxide on a Pd_4_Sn alloy nanowire (NW) prepared via two-step synthesis (Fig. 1) present efficient reactivity in the direct production of H_2_O_2_. This approach involves the synthesis of the surface-rough Pd_4_Sn nanowires (PdSn-NW, Sn:Pd molar ratio is 4) by a solvothermal method firstly (Supplementary Fig. 1)^32^. Then, metal Pd precursor was deposited onto the Pd_4_Sn nanowires, followed by dispersing on the TiO_2_ support. After rapid annealing of the material in air, an efficient catalyst for direct H_2_O_2_ synthesis was obtained and denoted as Pd_L_/PdSn-NW catalyst (Supplementary Fig. 2). The unsupported Pd_L_/PdSn-NW presents the morphology of worm-liked nanowires and some nanowires connect each other to form interconnected structures (Fig. 2a). The outmost surface of the structures contains Pd and Sn terminated with oxygen because both of the two metals were oxidized after the annealing in the air as shown in Fig. 1. We also synthesize and test the TiO_2_-supported PdSn nanowire catalyst prepared via one-step synthesis (denoted as PdSn-NW). As can be seen, the two unsupported materials show similar nanowire morphology (Fig. 2a and Supplementary Fig. 1). After loading onto TiO_2_, the nanowire morphologies do not change (see discussion below).

## Results and discussion

### Materials synthesis and catalytic performance

The catalytic reactivity of the TiO_2_-supported PdSn nanowire catalyst synthesized by the two-step (Pd_L_/PdSn-NW, 4.1 wt.%Pd loading) was evaluated in the direct synthesis of H_2_O_2_ at zero Celcius. In comparison, we also synthesized PdSn-NW catalyst via one-step (PdSn-NW), PdSn nanoparticle (PdSn-NP), and SnO_x_ supported on PdSn-NW (SnO_x_/PdSn-NW) via the two-step approach. The supports of all these reference catalysts are TiO_2_ and all the activity data reported in the work are achieved from catalysts supported on TiO_2_. Prior to dispersing on TiO_2_, the morphologies of these reference nanowire/nanoparticles are well characterized using TEM (Supplementary Figs. 3, 4). It should be mentioned that both TEM-EDS and X-ray photoelectron spectroscopy (XPS) analysis indicate that there is no residual Br ion detected on the Pd catalyst (Supplementary Fig. 5) and therefore, the effect of the Br ion in the H_2_O_2_ synthesis can be excluded. The supported Pd_L_/PdSn-NW catalyst synthesized by two-step shows the high H_2_O_2_ producibility of ~680 mol/kg h^−1^ after annealing at 350 °C in the air (Fig. 2b). The addition of Sn onto the PdSn nanowires leads to the formation of SnO_x_/PdSn-NW catalyst, which is less active than the supported Pd_L_/PdSn-NW catalyst after annealing at 350 °C in air. Moreover, with the increase of Sn loading (4.5–13 wt.%), the reactivity of the SnO_x_/PdSn-NW catalyst further decreases, indicating the addition of Sn on the PdSn nanowires has a negative effect on the reactivity of the PdSn-NW (Fig. 2b). However, the Pd_L_/PdSn-NW catalyst annealing at 350 °C presents the high hydrogenation activity, which is undesirable for the DHS^3^. We investigated the effect of the annealing temperature on the reactivity of the Pd_L_/PdSn-NW catalyst and found that annealing the Pd_L_/PdSn-NW catalyst at 400 °C presented the H_2_O_2_ producibility of ~528 mol/kg h^−1^ with the complete absence of hydrogenation (Fig. 2c). Therefore, 400 °C was employed as the annealing temperature for all supported catalysts in the following discussion, unless otherwise noted. Moreover, the Pd/Sn ratios and metal loadings of all the catalysts were measured by EDS and ICP-OES analysis, and a good agreement was obtained using the two techniques (Supplementary Figs. 6 and 7).

When using the two-step approach to deposit Pd precursor onto the as-synthesized PdSn nanowires to form Pd_L_/PdSn-NW catalyst, the Pd loading is precisely controlled to cover the PdSn nanowires as monolayer (Supplementary Note 1) and two layers, which are denoted as Pd_L_/PdSn-NW and Pd_2L_/PdSn-NW, respectively. The annealed Pd_L_/PdSn-NW catalyst shows superior reactivity than the PdSn-NW catalyst prepared via one-step (Fig. 2d and Supplementary Fig. 8). Although PdSn nanowire alone (PdSn-NW) shows no hydrogenation or decomposition activity (Fig. 2d), the H_2_O_2_ selectivity is only ~70% (Table 1). This is explained by the fact that the H_2_O_2_ selectivity is calculated from the first step in this two-step process (Supplementary Fig. 9). Furthermore, the annealed Pd_L_/PdSn-NW catalyst presents outstanding catalytic activity for H_2_O_2_ production and higher H_2_O_2_ selectivity, as compared to Pd_2L_/PdSn-NW catalyst (Fig. 2d). This indicates that the addition of the extra Pd onto the Pd_L_/PdSn-NW has a negative effect on the H_2_O_2_ production. Besides, both the H_2_O_2_ hydrogenation and decomposition are completely inhibited on the annealed Pd_L_/PdSn-NW catalyst, as compared to other active Pd catalysts reported. Furthermore, the annealed Pd_L_/PdSn-NW catalyst is very stable in the reaction and no deactivation is found in multiple runs (Supplementary Fig. 10). The reactivity of the supported Pd_L_/PdSn-NW catalyst was also compared with other Pd-based catalysts (Fig. 2d and Table 1, Supplementary Tables 1 and 2), such as Pd nanoparticles, PdSn nanoparticles, and nice PdSn nanowires prepared by the approach reported in the literature^32^. None of them showed comparable H_2_O_2_ producibility and low decomposition/hydrogenation activity (Supplementary Fig. 11) as the supported Pd_L_/PdSn-NW catalyst exhibiting the H_2_O_2_ selectivity of >95% under the same conditions. It should be mentioned that the H_2_O_2_ productivity slightly slows down over time (after 30 min) and it is partially ascribed to the decrease of the reactant gas concentration and the accumulation of H_2_O_2_ in the autoclave according to Le Chatelier’s principle (Supplementary Fig. 12). Besides, it possibly suggests that the catalyst changes in the presence of high concentration H_2_O_2_ and either become less active or begins to degrade H_2_O_2_ at high H_2_O_2_ concentrations.

**Table 1:** Catalytic performances of various Pd catalysts supported on TiO_2_ after pretreating under different conditions in the direct synthesis of H_2_O_2_ from H_2_ and O_2_

Entry	Catalyst^a^	Annealing^b^	H_2_O_2_ Prod.	H_2_O_2_	H_2_
			(mol/kg h^−1^)	Sel. (%)	Conv.(%)
1	PdSn-NW	400 °C, 8 min	389	70.6	22.1
2	Pd_L_/PdSn-NW	400 °C, 8 min	528	95.3	22.1
3	Pd_2L_/PdSn-NW	400 °C, 8 min	290	38.3	30.5
4	PdSn-NP	400 °C, 8 min	99	15.6	23.8
5	PdSn-NP	400 °C, 4 h	22	6.9	12.1
6	PdSn-NP	n.a.	72	5.8	47.9
7	Pd-NP	400 °C, 8 min	68	43.5	4.9
8	Pd-NP	n.a.	56	11.1	16.0

^a^All the catalysts are supported on TiO_2_.

^b^n.a.: no annealing.

### Materials characterization

**Fig. 3:**
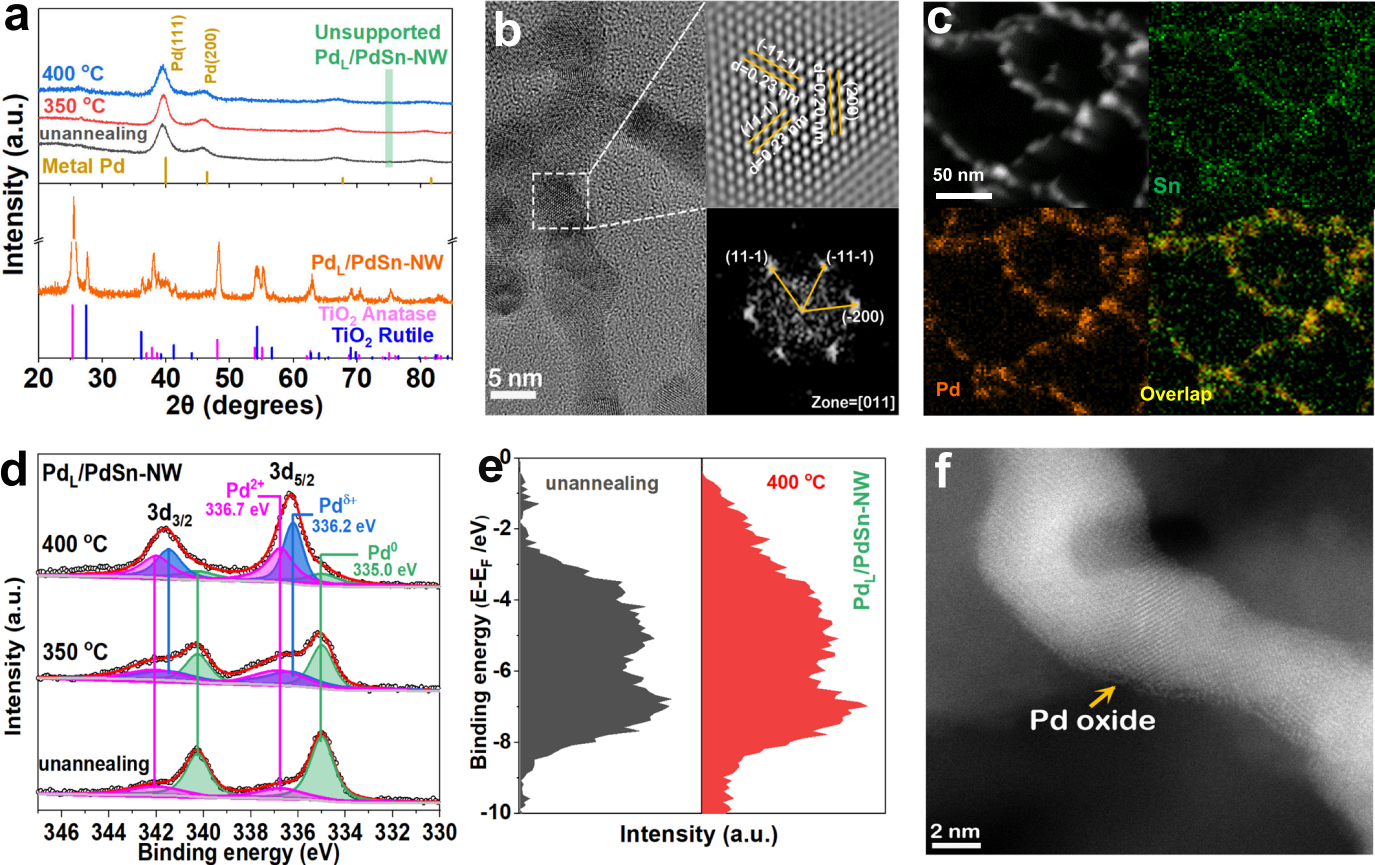
Structural characterization of the unsupported and supported Pd_L_/PdSn-NW catalysts before and after annealing in air at 400 °C. **a** XRD patterns of the unsupported and supported Pd_L_/PdSn-NW catalysts before and after annealing in air. **b** HRTEM image of the unsupported Pd_L_/PdSn-NW sample showing the lattice fringes of the PdSn phase. **c** STEM image and STEM-EDS mapping of the supported Pd_L_/PdSn-NW showing the close proximity of both Pd and Sn elements, indicating the successful synthesis of Pd_4_Sn alloy. **d** XPS spectra of Pd 3*d* core level of the supported Pd_L_/PdSn-NW catalyst before and after annealing in air. **e** Surface valence band photoemission spectra of the supported Pd_L_/PdSn-NW catalysts annealing at different temperatures in air. **f** HAADF-STEM image of the supported Pd_L_/PdSn-NW catalyst after annealing showing a layer of Pd oxide on the PdSn nanowires.

The composition and morphology of the Pd_L_/PdSn-NW catalyst are first characterized by X-ray diffraction (XRD) and electron microscopy. XRD patterns of the unsupported PdSn nanowire prepared by two-step before and after annealing show the diffraction peaks located at lower diffraction angles (i.e., larger d spacings) as compared to that of metal Pd (Fig. 3a), indicating the crystal expansion of metal Pd due to the alloying of Sn atom. Annealing this unsupported PdSn nanowire at 350 °C or 400 °C does not make significant changes on the XRD patterns, indicating that the nanowire is thermally stable. When dispersing the nanowire on TiO_2_, XRD pattern of the supported Pd_L_/PdSn-NW catalysts only presents the diffraction peaks of the support TiO_2_ (Fig. 3a and Supplementary Fig. 13), indicating the high dispersion of PdSn nanowire on the support. After annealing the supported Pd_L_/PdSn-NW catalyst at different temperatures (350 °C and 400 °C, Supplementary Fig. 13a) in air, we do not find the presence of other diffraction peak or phase separation, indicating that the PdSn-NW and Pd_L_/PdSn-NW catalysts are quite stable after the pretreatment. The representative high-resolution transmission electron microscopy (TEM)/scanning transmission electron microscopy (STEM) images of the Pd_L_/PdSn-NW are shown in Fig. 3b, c. The nanowires have the diameter ranging from 5 to 10 nm and STEM-EDS line scanning shows the presence of both Pd and Sn in the nanowire (Supplementary Fig. 14). The high-resolution TEM of the nanowire is shown in Fig. 3b and the image exhibits the lattice fringes of 0.23 and 0.20 nm, corresponding to the (111) and (200) plane of Pd_4_Sn phase, respectively (more images are shown in Supplementary Fig. 15). STEM-EDS mapping demonstrated that both Pd and Sn signals are uniformly dispersed in the nanowires (Fig. 3c). Therefore, the electron microscopy characterization of the annealed Pd_L_/PdSn-NW sample demonstrates that the nanowire morphology of PdSn-NW is well maintained after loading onto TiO_2_ and annealing in air.

XPS is used to investigate the surface species of the supported Pd_L_/PdSn-NW catalyst during the annealing process (Fig. 3d, e, Supplementary Figs. 16–19). As shown in Fig. 3d, the XPS spectrum of the unannealed Pd_L_/PdSn-NW catalyst shows that the main Pd species is Pd metal and the presence of a very small amount of Pd^2+^ prior to annealing. After annealing at 350 °C in air, the Pd^2+^ species content increases and Pd metal is still the dominant species on the surface. After annealing the Pd_L_/PdSn-NW at 400 °C in air, we observe that the main Pd on the catalyst is Pd^2+^ species with a small amount of Pd metal, demonstrating the oxidation of metal Pd after annealing. Furthermore, the XPS analysis indicates that the Sn species on the supported Pd_L_/PdSn-NW catalyst is SnO_x_ (Supplementary Fig. 19) and the Pd/Sn ratio is ~2. Since Sn/PdSn-NW catalysts are less active and selective than PdSn-NW catalyst, the active site is not ascribed to the SnO_x_. Therefore, the rapid annealing process generated Pd oxide on the PdSn nanowires, which is observed in the HAADF-STEM image (Fig. 3f and Supplementary Fig. 20). The d-band model has been widely used in order to understand activity trends in metal-surface-catalyzed reactions^33^. Here, the d-band center obtained from XPS was used to study the interaction between gas adsorption/intermediates and metal surface during annealing in air. The surface valence band photoemission spectrum of the annealed Pd_L_/PdSn-NW is more delocalized and widely distributed than that of the unannealed Pd_L_/PdSn-NW (Fig. 3e), indicating the different activation ability of gas molecules or intermediates on the annealed Pd_L_/PdSn-NW catalyst^34^. The annealed Pd_L_/PdSn-NW catalyst, therefore, provides Pd oxide to modulate the adsorption/desorption behaviors of the reactants on the catalyst surface, which was confirmed by the improved H_2_O_2_ producibility of Pd_L_/PdSn-NW (Table 1) and density functional theory (DFT) calculation below.

**Fig. 4:**
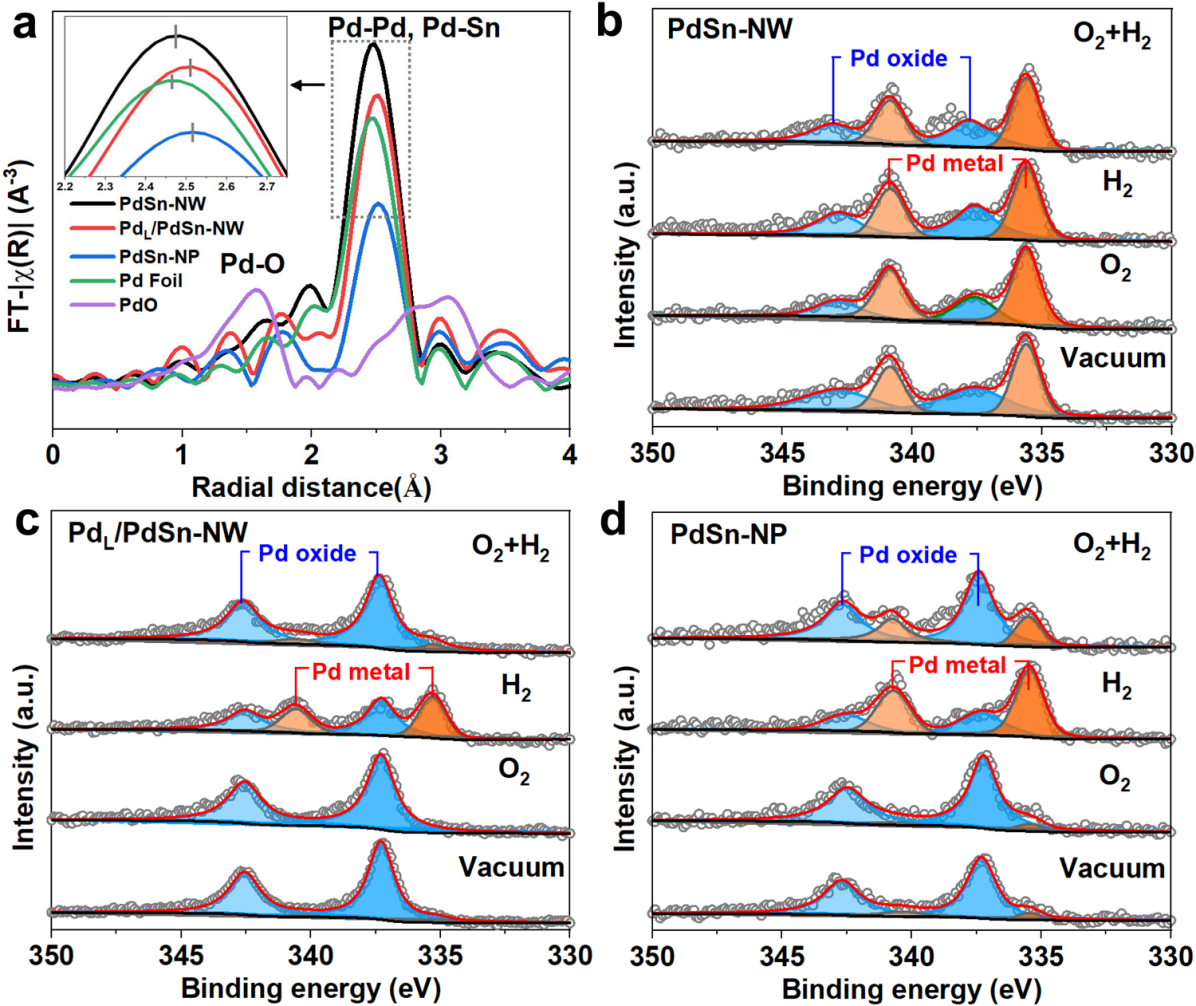
EXAFS spectra of the PdK edge of PdSn catalysts and reference samples, and NAP-XPS of Pd 3*d* spectra for PdSn catalysts under different treatment conditions. **a** EXAFS spectra of the PdK edge of PdSn catalysts after annealing in air. **b** In situ NAP-XPS of Pd 3*d* spectra for PdSn-NW in the presence of O_2_, H_2_, and O_2_/H_2_. **c** In situ NAP-XPS of Pd 3*d* spectra for Pd_L_/PdSn-NW after annealing in air in the presence of O_2_, H_2_ and O_2_/H_2_. **d** In situ NAP-XPS of Pd 3*d* spectra for PdSn-NP after annealing in air in the presence of O_2_, H_2_ and O_2_/H_2_.

The chemical environment of the Pd atoms in the supported Pd_L_/PdSn-NW catalyst after annealing in the air was examined via X-ray adsorption spectroscopy (Fig. 4a and Supplementary Figs. 21 and 22). We also performed the XAS measurements of the Pd foil, PdO, PdSn-NW, and PdSn-NP catalysts for comparison. For the reference Pd foil, a major peak attributing to the Pd-Pd scattering was observed. The EXAFS spectra of PdSn-NW and PdSn-NP catalysts show a similar or slightly expanded scattering distance as compared to that of the metal Pd foil (Fig. 4a), which is attributed to the Pd-Pd and PdSn scattering. The Pd-K edge EXAFS of the supported Pd_L_/PdSn-NW catalyst after annealing (air, 400 °C) also have a major peak corresponding to Pd-Pd and PdSn scattering (Fig. 4a), indicating the main Pd phase on the Pd_L_/PdSn-NW catalyst is metal Pd and the rapid annealing process does not change the bulk phase of the PdSn nanowires. EXAFS fit gave a coordination number of 4.2 at a bond distance of 2.76 Å (Supplementary Table 3). The EXAFS is fitted to Pd-Pd and PdSn scattering features because the XRD results clearly indicate that the bulk phase of the PdSn nanowire after annealing in air is metal Pd (Fig. 3a) and both XRD and XAS are bulk techniques. Furthermore, the high-resolution TEM (HRTEM) image shows the lattice fringes corresponding to metallic PdSn nanowire (Supplementary Fig. 15a). Since XPS is more surface sensors for observing the changes under different conditions than XRD and XAS, We performed near-ambient pressure X-ray photoelectron spectroscopy (NAP-XPS) to in-situ investigate the surface properties of the PdSn catalysts (both PdSn nanoparticle and PdSn nanowire prepared via one-step and two-step approaches) in the flowing O_2_, H_2_ and O_2_/H_2_ mixture (Fig. 4b–d). Conventional PdSn nanoparticle catalyst showed the presence of Pd oxides in both vacumm and the flowing O_2_, whereas metal Pd was formed after switching to H_2_ or H_2_/O_2_ mixture (Fig. 4d), indicating that the conventional PdSn nanoparticles are not stable in H_2_ and H_2_/O_2_ atmospheres. For the PdSn nanowire catalysts, the Pd_L_/PdSn-NW prior to annealing mainly shows the metal Pd phase on the catalyst surface (Fig. 4b) and there is a very small change for the Pd 3*d* XPS spectra of the catalyst after flushing in H_2_, O_2_, and H_2_/O_2_, indicating that the unannealing Pd_L_/PdSn-NW maintain the metal Pd surface in the flowing H_2_/O_2_. Likewise, the Pd 3*d* XPS spectrum of the Pd_L_/PdSn-NW after annealing in air at 400 °C indicated that Pd oxide is the main Pd species on the surface at vacuum (Fig. 4c). The introduction of a flow of O_2_ has no observed effect on the Pd 3*d* XPS spectrum of the annealed Pd_L_/PdSn-NW. After introducing a flow of H_2_, we observed the presence of metal Pd, as well as the presence of Pd oxide. However, in the presence of H_2_/O_2_ mixture, the Pd_L_/PdSn-NW surface after annealing was maintained as Pd oxide. Therefore, in-situ NAP-XPS evidenced that the surface species of the Pd_L_/PdSn-NW after annealing is Pd oxide in the reaction feed of H_2_/O_2_, while the major Pd species on both PdSn-NP and PdSn-NW are metal Pd in the presence of H_2_/O_2_.

### Theoretical studies on reaction mechanism

**Fig. 5:**
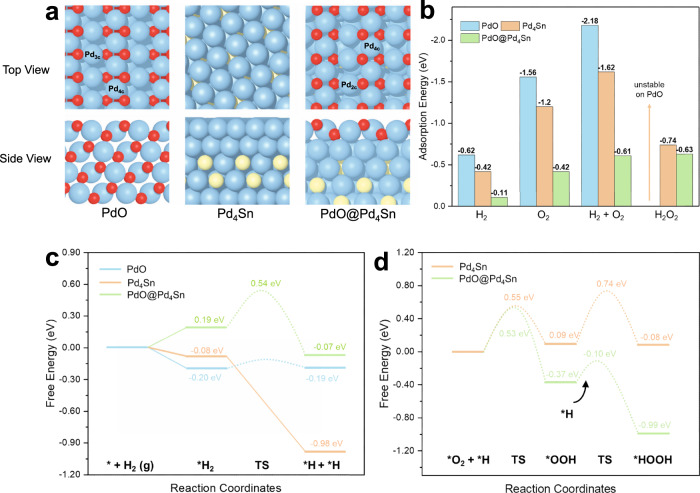
Adsorption energy of key species and proposed mechanism. **a** DFT optimized structures of PdO(101), Pd_4_Sn, and PdO@Pd_4_Sn with **b** adsorption energies of H_2_, O_2_, H_2_ + O_2_, and H_2_O_2_, **c** free energy profiles for H_2_ activation, and **d** free energy profiles for O_2_ reduction by the surface hydrides on these three models. TS transition state. Color code: Pd, blue; O, red; H, white; Sn, yellow.

Based on the above reactivity and characterization results, we propose that the excellent reactivity of the PdSn nanowires prepared by the two-step approach (Pd_L_/PdSn-NW) is associated with the Pd oxide layer on PdSn nanowire which was detected via AC-STEM and NAP-XPS (Figs. 3 and 4). The stable Pd oxide layer on PdSn nanowire in the presence of H_2_/O_2_ exhibited enhanced reactivity as compared to the conventional PdSn nanoparticle and PdSn nanowire prepared by a one-step approach. To better understand the unique properties of the Pd oxide layer on the Pd_L_/PdSn-NW in direct H_2_O_2_ synthesis, we performed DFT calculations based on three models of PdO(101), Pd_4_Sn and PdO monolayer supported on Pd_4_Sn(111) (PdO@Pd_4_Sn) (Fig. 5a). The PdO(101) surface contains tetra-coordinated Pd (denoted as Pd_4c_) and tri-coordinated Pd (denoted as Pd_3c_) and the outmost surface atoms of the Pd_4_Sn model are Pd atom, while the PdO@Pd_4_Sn surface contains Pd_4c_ and bi-coordinated Pd (denoted as Pd_2c_). Details for model construction can be found in the computational method section. Bader charge analysis shows that Pd_4c_ on PdO@Pd_4_Sn and PdO(101) are charged similarly with +0.83|e| and +0.85|e| (Supplementary Fig. 23), respectively. Nevertheless, the charges of Pd_2c_ on PdO@Pd_4_Sn and Pd_3c_ on PdO(101) are different, with the former being charged +0.46|e| and the latter having a charge of +0.67|e|. The surface Pd atoms in Pd_4_Sn are slightly positive by +0.11|e|, which is therefore more metallic than those in PdO(101) and PdO@Pd_4_Sn. Thus, these three catalysts with different Pd chemical states may lead to different interactions between the adsorbate and the catalyst.

Subsequently, we investigated the adsorption behavior of the reaction molecules (O_2_, H2, and H_2_O_2_) on PdO(101), Pd_4_Sn, and PdO@Pd_4_Sn. The adsorption configurations and corresponding adsorption energies are shown in Supplementary Fig. 24 and Fig. 5b, respectively. The calculation results show that all three molecules exhibit strong adsorption on the PdO(101) surface. H_2_ prefers to adsorb on top of Pd_3c_ and O_2_ tends to adsorb on the Pd_3c_-Pd_3c_ bridge site, leading to adsorption energies of −0.62 and −1.56 eV, respectively. For H_2_O_2_, we found that it has a spontaneous dissociation to form two OHs on the two Pd_3c_ sites on PdO(101), suggesting that PdO(101) has poor selectivity for H_2_O_2_ synthesis. For PdO@Pd_4_Sn, its binding to the three molecules is significantly weaker than that on PdO(101). H_2_ is physically adsorbed on the Pd_2c_ top sites (2.76 Å away from Pd_2c_) with an adsorption energy of −0.11 eV, the adsorption of O_2_ on the Pd_2c_-Pd_2c_ bridge sites leads to small adsorption energy of −0.42 eV. H_2_O_2_ adsorbs on Pd_2c_-Pd_2c_ bridge sites, yielding moderate adsorption energy of −0.63 eV. Likewise, as for the Pd_4_Sn surface, H_2_ is adsorbed on the top of Pd while the O_2_ molecule connects with four surface Pd atoms, having the adsorption strengths as those located on between PdO(101) and PdO@Pd_4_Sn. To gain in-depth insight into the relation between electronic structure and catalytic behavior, the projected density of states (PDOSs) of the surface Pd- ${d}_{{z}^{2}}$ states on PdO(101) and PdO@Pd_4_Sn were calculated and compared since the ${d}_{{z}^{2}}$ states are considered to play an important role in molecule adsorption^35–37^. As can be seen in Supplementary Fig. 25, the distribution of ${d}_{{z}^{2}}$ of surface Pd_3c_ on PdO is more localized than that of surface Pd_2c_ on PdO@Pd_4_Sn. Furthermore, a sharp peak of ${d}_{{z}^{2}}$ states exists across the Fermi level on PdO(101), implying strong interaction between surface Pd and the reaction species via donating/accepting electrons. In contrast, on PdO@Pd_4_Sn, the distribution of Pd-${d}_{{z}^{2}}$ state is less significant around the Fermi level, indicating the weaker binding of reaction species to Pd. It is therefore proposed that different adsorption behaviors of O_2_, H_2_, and H_2_O_2_ on the three models lead to different catalytic performances in the direct synthesis of H_2_O_2_.

In the following, we investigated the reaction mechanism of the O_2_ reduction on PdO(101), Pd_4_Sn, and PdO@Pd_4_Sn. First, we calculated the activation of the most stable adsorption state of H_2_, whose reaction pathways on these three models are shown in Fig. 5c. On PdO(101), H_2_ dissociation occurs readily with an energy barrier of 0.09 eV. In the final state, the two dissociated H atoms are adsorbed on the Pd_3c_-Pd_3c_ bridge site and the Pd_3c_ top site, respectively. In addition, it is found that the migration of H between two adjacent Pd_3c_ sites is facile with a small energy barrier of 0.11 eV, while the migration of H from the Pd_3c_ site to an adjacent oxygen site is difficult, requiring an energy barrier of 0.87 eV(Supplementary Fig. 26). It indicates that Pd-H hydride species are stable on PdO(101). On Pd_4_Sn, H_2_ activation on Pd_4_Sn is barrierless, similar to the previous DFT findings on Pd(111) surface^38^ (Supplementary Fig. 27). On PdO@Pd_4_Sn, the cleavage of H-H bond in the most stable H_2_ adsorbed has to overcome an energy barrier of 0.35 eV. Similar to the case on PdO(101), the Pd-H species is also stable because the energy barrier for H migration between Pd_2c_ sites (0.69 eV) is lower than that (1.07 eV) of its migration to oxygen sites (Supplementary Fig. 28). Therefore, H_2_ readily dissociates on all of PdO(101), Pd_4_Sn, and PdO@Pd_4_Sn to produce stable Pd-H hydride, which provides a hydrogen source for the subsequent O_2_ reduction (Fig. 5d). We also calculated other possible H_2_ dissociation pathways but they possess higher energy barriers (Supplementary Figs. 29 and 30). Then, we investigate the O_2_ reduction to H_2_O_2_ (energy profile in Fig. 5d and configurations in Supplementary Figs. 31 and 32). On the Pd_4_Sn, the energy barriers of the first (*O_2_ + *H → *OOH) and second (*OOH + *H → *H_2_O_2_) hydrogenation of O_2_ is 0.55 eV and 0.65 eV with the reaction energies of 0.09 eV and −0.01 eV, respectively. On PdO@Pd_4_Sn, the *O_2_ + *H → *OOH reaction encounters an energy barrier of 0.53 eV, while the subsequent *OOH + *H → *H_2_O_2_ process has a lower energy barrier of 0.27 eV. This result indicates that the reaction of O_2_ reduction to produce H_2_O_2_ has a lower rate-determining energy barrier on PdO@Pd_4_Sn than that on Pd_4_Sn, in agreement with the experimental observations that PdO@Pd_4_Sn has better catalytic performance than Pd_4_Sn prepared by one-step. We did not infer the reaction pathway on PdO(101) because H_2_O_2_ was proved to be unstable on this surface (Fig. 5b and Supplementary Fig. 25), leading to low H_2_O_2_ production.

In addition, we simulated three main side reactions of *O_2_, *OOH, and *H_2_O_2_ dissociation on Pd_4_Sn and PdO@PdSn, and the corresponding structures of IS, TS, and FS are shown in Supplementary Figs. 33 and 34. On Pd_4_Sn(111), the *O_2_ and *OOH dissociations are barrierless while the *HOOH dissociation has a small barrier of 0.07 eV (Supplementary Fig. 33), suggesting the poor selectivity of H_2_O_2_ on Pd_4_Sn(111). On PdO@Pd_4_Sn, the energy barriers of the three side reactions are 1.74, 0.53, and 0.32 eV (Supplementary Fig. 34), respectively, which are larger than those of the corresponding competitive reactions (0.53, 0.27, and 0.14 eV for O_2_ hydrogenation, OOH hydrogenation, and H_2_O_2_ desorption, respectively, Supplementary Figs. 32, 34). Therefore, these results suggest the high selectivity of H_2_O_2_ on PdO@Pd_4_Sn, which is consistent with the experimental observations (Fig. 2d and Table 1).

Selective production of H_2_O_2_ is a challenge in the direct H_2_O_2_ synthesis (DHS) from H_2_ and O_2_. In this contribution, we develop a two-step approach to prepare PdSn nanowires to efficiently catalyze direct H_2_O_2_ synthesis (DHS). This approach involves the first synthesis of the surface-rough Pd_4_Sn nanowire (PdSn-NW) by a solvothermal method. Then Pd precursor was deposited onto the PdSn alloy nanowire (NW), followed by annealing in air. The as-prepared Pd_L_/PdSn-NW via the two-step approach presents efficient reactivity with H_2_O_2_ producibility of >520 mol kg_cat_^−1^ h^−1^ and selectivity of >95% in the direct production of H_2_O_2_ at zero Celcius. For the PdSn nanowire prepared by one-step method and PdSn nanoparticle catalysts, the catalyst surfaces are prone to be reduced in the flowing H_2_/O_2_ and show low H_2_O_2_ reactivity.

The excellent H_2_O_2_ production over the Pd_L_/PdSn-NW catalyst is attributed to the presence of Pd oxide layer on the PdSn nanowires. The Pd oxide layer is stable against reduction or oxidation in the flowing H_2_/O_2_, while other PdSn nanoparticles and PdSn nanowire catalysts undergo a reduction in the presence of H_2_/O_2_. The layered Pd oxide enables less adsorption of oxygen/hydrogen and decreases the rupture of both O-O and H-H bonds, as well as less adsorption of peroxide produced, leading to the complete inhibition of the H_2_O_2_ hydrogenation and decomposition. Therefore, engineering the surface of PdSn nanowires via two-step approach can generate a layer of Pd oxide on the PdSn nanowire, presenting excellent reactivity in the direct H_2_O_2_ synthesis, which provides a promising strategy to design and develop highly active DHS catalyst.

## Methods

### Chemicals

Bis (acetylacetonato) palladium (II) (Pd(acac)_2_, 99%), tin(II) acetate (Sn(Ac)_2_, 95%), methanol (CH_3_OH, HPLC grade), titanium dioxide (TiO_2_, P25, 99%) were purchased from Sigma-Aldrich. Palladium(II) nitrate dihydrate (Pd(NO_3_)_2_·2H_2_O, 99%), polyvinylpyrrolidone (PVP, MW = 58000), Tin(IV) chloride pentahydrate (SnCl_4_·5H_2_O, 99%) were purchased from J&K Scientific Ltd. Ethylene glycol (EG, analytical grade), N,N-Dimethylacetamide (DMAC, analytical grade), ammonium bromide (NH_4_Br, analytical grade) were purchase from Sinopharm Chemical Reagent Co. Ltd.(Shanghai, China). The de-ionized water (DI H_2_O,18 MΩ/cm) used in all experiments was obtained by passing through an ultra-pure purification system. All the chemicals were used without further purification.

### Synthesis of unsupported PdSn nanowires via one-step

In a typical synthesis, the PdSn NWs (Pd_4_Sn, marked as PdSn) were synthesized according to our previous report^32^. Briefly, 7.6 mg of Pd(acac)_2_, 1.5 mg of Sn(Ac)_2_, 15 mg of NH_4_Br, 100 mg of PVP, 2 mL of DMAC, and 8 mL of EG were added into a vial (30 mL). And then, the mixture was sonicated for 15 min to ensure form a homogeneous solution. The solution was heated and maintained at 180 °C for 2 h in an oil bath. After that, the mixture was cooled down to room temperature. Finally, the precipitate was centrifuged and washed with ethanol/acetone mixture.

### Synthesis of unsupported Pd_x_/PdSn-NW and Sn_y_/PdSn-NW via two-step

In a typical synthesis, the Pd_x_/PdSn or Sn_y_/PdSn NWs were synthesized by the following steps. Briefly, 7.6 mg of Pd(acac)_2_, 1.5 mg of Sn(Ac)_2_, 15 mg of NH_4_Br, 100 mg of PVP, 2 mL of DMAC, and 8 mL of EG were added into a vial (30 mL). Then, the mixture was sonicated for 15 min to ensure form a homogeneous solution. The solution was heated and maintained at 180 °C for 2 h in an oil bath. After that, the mixture was cooled down to room temperature to achieve the unsupported PdSn nanowires. The role of NH_4_Br in the synthesis of these worm structures is to induce the formation of the PdSn nanowires. Subsequently, the desired amount of Pd(acac)_2_ or Sn(Ac)_2_ solution (including 320 μL of EG and 80 μL of DMAC, e.g. 1.9 mg Pd(acac)_2_) was added into the above solution. Next, the mixture was continuously stirred and held for 30 min at room temperature, followed by heating to 150 °C (hold for 2 h) with vigorous stirring. Then, the mixture was cooled down to ambient temperature again. Finally, the precipitate was centrifuged and washed with ethanol/acetone mixture. The precipitate achieved with the addition of 1.9 mg Pd precursor in the second step is denoted as unsupported Pd_L_/PdSn-NW and the sample achieved with the addition of Sn(Ac)_2_ solution in the second step is denoted as unsupported SnO_x_/PdSn-NW.

### Synthesis of unsupported Pd nanoparticles

The unsupported Pd NPs were synthesized from a procedure similar to that of PdSn nanowires synthesized via one-step, but without the addition of Sn(Ac)_2_.

### Synthesis of unsupported PdSn nanoparticles (PdSn-NP)

The bimetallic PdSn NP catalysts were synthesized according to a previous report^3^. In brief, 62.5 mg of Pd(NO_3_)_2_·2H_2_O, 2 mL of DI water were added into a vial (30 mL). The solution was heated to 80 °C with continuous stirring. Then, 1 mL of DI water solution (including 73.8 mg of SnCl_4_·5H_2_O) was added to the vial and held for 15 min under stirring. Next, 0.6 g of TiO_2_ and 1 mL of DI water were added to the solution, and the mixture was heated to 110 °C with continuous stirring in the air to evaporate the water. Subsequently, the solid sample was placed in a tube furnace and heated to 500 °C for 3 h under static air (heating rate of 10 °C/min). After cooling down, the solid sample was further heated to 200 °C for 2 h in a flow of 5% H_2_/Ar (heating rate of 10 °C/min). Then, the solid sample was annealed in static air at 400 °C for 8 min or 4 h, respectively (heating rate of 10 °C/min). The resultant was denoted as PdSn-NP.

### Synthesis of catalysts supported on TiO_2_

Typically, the commercial TiO_2_ material (anatase) and 6 mL of CH_3_Cl were added into a 30 mL vial. The PdSn-NW or nanoparticle materials were firstly dispersed in 6 mL of CH_3_Cl and ultrasonicated for about 5 min. The resulting mixture was added to the TiO_2_ vial. Next, the mixture was continuously ultrasonicated for 30 min. Finally, the products were collected by centrifugation and dried at 60 °C overnight.

### Annealing treatment of the supported catalysts

The as-synthesized catalysts were treated via a rapid annealing process. Firstly, the tube furnace was heated to 400 °C and held for 30 min under static air. Next, the appropriate amount of catalyst was placed in the porcelain boat and pushed into the tube furnace. The catalyst left for several minutes by annealing and was taken out (8 min) for cooling. The final products were denoted as Pd_L_/PdSn-NW (supported on TiO_2_, the “NW” represents nanowires) and collected for further characterization.

### Catalyst characterization

The crystallographic structure of all the samples was determined by Powder XRD (Rigaku Ultima IV, Japan) patterns using a Cu Kα X-ray source (*λ* = 1.54056 Å). The morphology of all the samples was imaged by transmission electron microscope (TEM, JEM-1400, JEOL Co., Japan). The HRTEM, line-scan analysis, and elemental mapping were carried out on FEI Tecnai F30 electron microscope with an acceleration voltage of 200 kV. STEM images were obtained on an FEI Titan Themis Z, operating at 60-300 kV to observe the surface morphology of the materials. Before microscopy examination, the catalyst powders were ultrasonically dispersed in ethanol and then a drop of the solution was put onto a carbon-coated copper grid. The elemental content of all the samples was examined by scanning electron microscopy energy dispersive spectrometer (SEM-EDS, ZEISS Sigma, Germany) and inductively coupled plasma-atomic emission spectrometry (ICP-AES, iCap 7000, USA). The chemical compositions and valence states of all the samples were analyzed by XPS (K-Alpha, USA). NAP-XPS (SPECS Surface Nano Analysis GmbH, Germany) measurements were carried out on a SPECS system equipped with a differentially pumped Phoibos hemispherical electron energy analyzer using monochromatic Al Kα radiation (1486.6 eV). The H_2_ or O_2_ (99.999%) flow was introduced into the NAP cell and the total pressure was kept constant at 0.2 mbar via the electronic back-pressure regulator. X-ray Absorption Fine Structure (XAFS) of all samples was analyzed by TLS-01C beamline of the National Synchrotron Radiation Research Center (NSRRC, Hsinchu, Taiwan), and data were processed according to standard procedures using the Demeter program package (Version 0.9.24)^39^.

### Direct H_2_O_2_ synthesis

All the catalysts supported on TiO_2_ were tested in the direct H_2_O_2_ synthesis. Direct H_2_O_2_ synthesis experiment was carried out in a stainless-steel semibatch autoclave with a volume of 50 mL. Briefly, 5 mg of supported catalyst, 5.54 g of MeOH, and 3.0 g of H_2_O (both HPLC grade) were added to the autoclave. Then purged three times with O_2_ (0.4 MPa) to the autoclave, and with filled with O_2_ (0.4 MPa) and 5% H_2_/Ar (3.6 MPa) until the total pressure of 4.0 MPa at room temperature. All experiments were performed in an ice-water bath and kept continuous stirring (1200 rpm) of reaction time for 15 min. The reaction was carried out at zero Celsius because it is much safer and easier to carry out in the lab. We also investigated the reactivity of the catalyst at room temperature for comparison. The producibility of the Pd_L_/PdSn-NW catalyst at room temperature is slightly lower than that performed at zero Celcius in the direct H_2_O_2_ synthesis (Supplementary Fig. 35). Control experiments are also performed by varying the stirring rate from 100-1200 rpm, and varying the catalyst mass from 3 to 10 mg using autoclaves having different volumes (50 mL and 100 mL). All these experiments show similar H_2_O_2_ producibility under these conditions. Therefore, it indicates that there are no interphase mass transfer constraints in the reaction under the present conditions (Supplementary Fig. 36).

### Quantification of gas product

The gas products were detected via a gas chromatograph (GC, 8890, Agilent) equipped with a thermal conductivity detector (TCD) and a MolSieve 5 A packing column (G3591-80022). Typically, after the direct H_2_O_2_ synthesis process, the autoclave was directly connected into the GC. The gas products were purged into GC by Ar.

The H_2_ conversion was calculated as follows:

1 $${{{{{{\rm{H}}}}}}}_{2}{{{{{\rm{conversion}}}}}}=\frac{n{({H}_{2})}_{{in}}\,-\,n{({H}_{2})}_{{out}}}{n{({H}_{2})}_{{in}}}\times 100\%$$

### H_2_O_2_ productivity evaluation

The H_2_O_2_ productivity was analyzed by titrating with acidified Ce(SO_4_)_2_ (0.05 M) and using ferroin (about 100 µL) as indicator^3,40^. The acidified Ce(SO_4_)_2_ solutions were standardized against (NH_4_)_2_Fe(SO_4_)_2_·6H_2_O using ferroin as an indicator. The H_2_O_2_ productivity and H_2_O_2_ selectivity were calculated based on the equations below:

$${{{{{{\rm{H}}}}}}}_{2}{{{{{{\rm{O}}}}}}}_{2}+{2{{{{{\rm{Ce}}}}}}}^{4+}\to {2{{{{{\rm{Ce}}}}}}}^{3+}+{2{{{{{\rm{H}}}}}}}^{+}+{{{{{{\rm{O}}}}}}}_{2}$$

2 $${{{{{{\rm{H}}}}}}}_{2}{{{{{{\rm{O}}}}}}}_{2}{{{{{\rm{selectivity}}}}}}=\frac{n{({H}_{2}{O}_{2})}_{{out}}}{n{({H}_{2}{O}_{2})}_{{out}}+n{({H}_{2}O)}_{{out}}}\times 100\%$$

3 $${{{{{\rm{Where}}}}}}\,{{{{{{\rm{n}}}}}}({{{{{{\rm{H}}}}}}}_{2}{{{{{\rm{O}}}}}})}_{{{{{{\rm{out}}}}}}}={{{{{{\rm{n}}}}}}({{{{{{\rm{H}}}}}}}_{2})}_{{{{{{\rm{converted}}}}}}}-{{{{{{\rm{n}}}}}}({{{{{{\rm{H}}}}}}}_{2}{{{{{{\rm{O}}}}}}}_{2})}_{{{{{{\rm{out}}}}}}}$$

### H_2_O_2_ hydrogenation and decomposition

H_2_O_2_ hydrogenation and decomposition were carried out using similar procedures to direct H_2_O_2_ synthesis. Typically, for H_2_O_2_ hydrogenation test (H_2_O_2_ + H_2_ → 2H_2_O), 5 mg of supported catalyst (support is TiO_2_), 5.54 g of MeOH, 2.0 g of H_2_O and 1.1 g H_2_O_2_ (30%) were added in the autoclave. Then, 3.6 MPa of H_2_/Ar (5% H_2_) was filled into the autoclave. Afterward, the reaction time extended until 30 min. For the H_2_O_2_ decomposition test (H_2_O_2_ → H_2_O + O_2_), 5 mg of catalyst, 5.54 g of MeOH, 2.0 g of H_2_O, and 1.1 g H_2_O_2_ (30%) were added to the autoclave. Then, 3.6 MPa of N_2_ was filled into the autoclave. Subsequently, the reaction was carried out for 30 min. The H_2_O_2_ hydrogenation or decomposition rate was calculated based as follows:

4 $${{{{{{\rm{H}}}}}}}_{2}{{{{{{\rm{O}}}}}}}_{2}{{{{{\rm{hydrogenation}}}}}}/{{{{{\rm{decomposition}}}}}}=\frac{n{({H}_{2}{O}_{2})}_{{in}}-n{({H}_{2}{O}_{2})}_{{out}}}{{W}_{{catalyst}}\times t}\times 100\%$$

### Computational details

All spin-polarized DFT calculations were carried out by using the Vienna Ab initio Simulation Package. Interactions between ion cores and valence electrons were described by the projected augmented wave method and the exchange-correlation function was by the Perdew–Burke–Ernzerhof functional based on generalized gradient approximation^41^. Wavefunctions were expanded by the plane wave basis with a cutoff energy of 400 eV^42^. The van der Waals interactions were included via DFT-D2 in Grimme’s scheme^43^. Transition states were determined by the Climbing Image Nudged Elastic Band and Dimer method^44,45^, and charge states were obtained from Bader Charge analysis^46^. For all models (crystallographic information files of Pd_4_Sn, PdO@Pd_4_Sn, and PdO(101) were provided in supplementary materials as Supplementary Data 1–3, respectively), Monkhorst–Pack mesh with 2 × 2 × 1 grid was adopted to sample the Brillouin zone^47^. Convergence criteria for structural optimizations were set to 10^−5^ eV and 0.02 eV/Å for energy and force, respectively. And for transition states searching, the criterion for force was set to 0.05 eV/Å. The (2 × 3)-PdO(101) surface was cleaved from the optimized PdO unitcell (*a* = *b* = 3.056 Å, *c* = 5.381 Å, in agreement with experimental values (*a* = *b* = 3.043 Å and *c* = 5.335 Å)), containing 4 O-Pd-O tri-atomic layers. The bottom two layers were fixed while the other layers with surface species were fully relaxed in structural optimization. The lengths of *a* and *b* of supercell are 12.38 and 9.17 Å, respectively. Since the Pd unit is composed of 4 Pd atoms, uniform Pd_x_Sn_y_ can only be constructed as Pd_2_Sn_2_ and Pd_3_Sn. In addition, our experiments show that nanowires expose more Pd on the surface, we, therefore, simulate the slab with 4 layers of uniform Pd_3_Sn covered with one layer of Pd (Supplementary Fig. 37). Then, the total Pd/Sn ratio equals 4:1. For all slabs, a vacuum space of 15 Å is adopted along the *z*-direction to avoid the interaction between periodic images.

The Gibbs free energy (*G*) is obtained by VASPKIT code^48^ via the following formula:

5 $$G=E+{E}_{{ZPE}}+{\varDelta U}_{0\to T}-T\times S$$

in which *E* is the electronic energy of DFT calculations, *E*_*ZPE*_ and *S* are the zero-point energy and entropy computed from vibrational frequency analysis. T was the temperature adopted in our experiment, 273.15 K. *ΔU*_*0→T*_ is the internal energy difference between 0 and T K.

## Supplementary information

Supplementary Information

Peer Review File

Description of Additional Supplementary Files

Supplementary Data 1

Supplementary Data 2

Supplementary Data 3

## Data Availability

All data needed to evaluate the conclusions in the paper are present in the paper and/or the Supplementary Information. The raw data sets used for the presented analysis within the current study are available from the corresponding authors on reasonable request. Source data are provided with this paper.

## Source data

Source Data

## Code availability

The code that supports the findings of this study is available from the corresponding author upon reasonable request.
