# Supplementary material for: Layered Pd oxide on PdSn nanowires for boosting direct H2O2 synthesis
Source: Nat Commun. 2022 Oct 14;13:6072. doi: 10.1038/s41467-022-33757-0 (PMC9568611; doi:10.1038/s41467-022-33757-0)
Supplement: Supplementary file 1 — Supplementary Information [file 41467_2022_33757_MOESM1_ESM.pdf]

Supporting Information for

Layered Pd oxide on PdSn Nanowires for Boosting Direct H_2O_2 Synthesis

Hong-chao Li,[1,2+] Qiang Wan[3+], Congcong Du,[1,2] Jiafei Zhao,[1,2] Fumin Li,[1,2] Ying Zhang,[1,2] Yanping Zheng,[1] Mingshu Chen,[1] Kelvin H.L. Zhang,[1,2] Jianyu Huang,[4] Gang Fu,[1,2] Sen Lin,[3*] Xiaoqing Huang[1,2*] and Haifeng Xiong[1,2,*]

[1] The State Key Laboratory of Physical Chemistry of Solid Surfaces, iChEM (Collaborative Innovation Center of Chemistry for Energy Materials), Department of Chemistry, College of Chemistry & Chemical Engineering, Xiamen University, Xiamen 361005, China

[2] Innovation Laboratory for Sciences and Technologies of Energy Materials of Fujian Province (IKKEM), 4221 Xiangan South Road, Xiamen 361102, P. R. China

[3] State Key Laboratory of Photocatalysis on Energy and Environment, College of Chemistry, Fuzhou University, Fuzhou 350002, China

[4] Clean Nano Energy Center, State Key Laboratory of Metastable Materials Science and Technology, Yanshan University, Qinhuangdao 066004, China

[+] These authors contribute equally

*To whom correspondence should be addressed.

E-mail: slin@fzu.edu.cn (S. Lin); hxq006@xmu.edu.cn (X. Huang); haifengxiong@xmu.edu.cn (H. Xiong)

Supplementary Figures

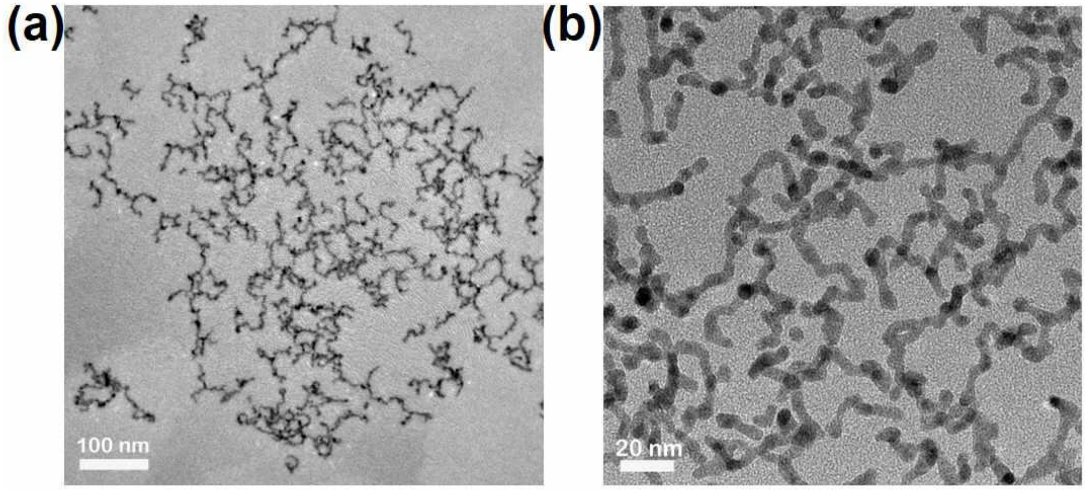

Supplementary Figure 1. | Morphology of the unsupported PdSn-NW. (a) TEM image and (b) Enlarged TEM image of the unsupported PdSn-NW catalyst. The scale bar in the a and b is 100 nm and 20 nm, respectively. These TEM images show the morphologies of the nanowires prior to loading on TiO_2 support.

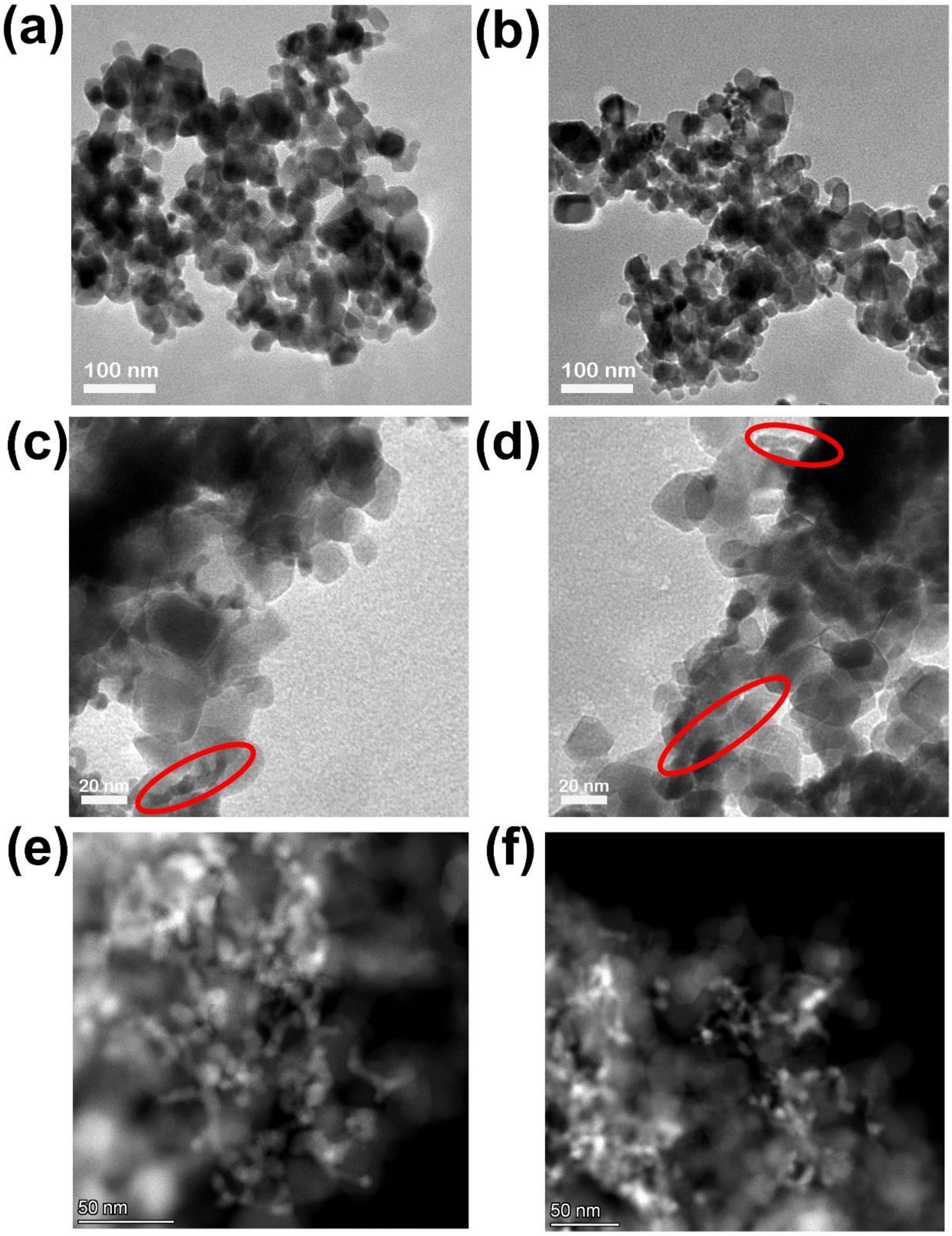

Supplementary Figure 2. | Morphology of the Pd_L/PdSn-NW. (a-d) TEM and (e-f) STEM images of the supported Pd_L/PdSn-NW catalyst. The support is TiO_2 and the red cycle shows the nanowires.

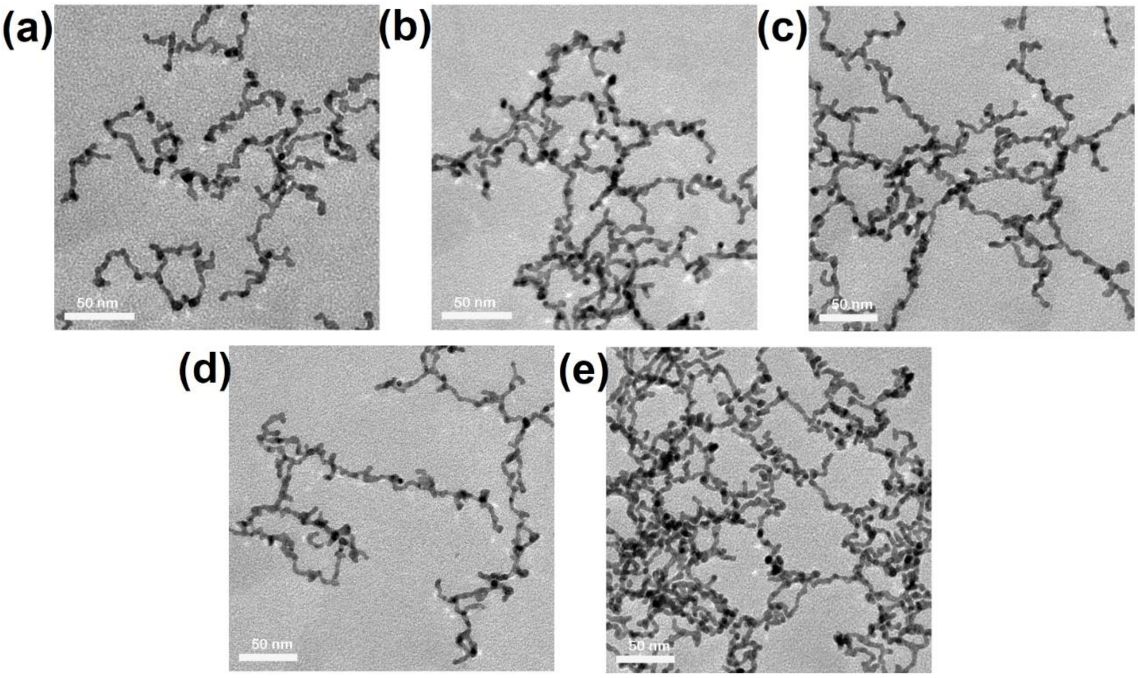

Supplementary Figure 3. | Morphology of the Pd catalysts. TEM images of unsupported Pd catalysts. (a) Pd_L/PdSn-NW, (b) Pd_{2L}/PdSn-NW, (c) $Sn_{4.5}$/PdSn-NW, (d) Sn_9/PdSn-NW, and (e) $Sn_{13.5}$/PdSn-NW. The scale bars are 50 nm. These TEM images show the morphologies of the nanowires prior to loading on TiO_2 support.

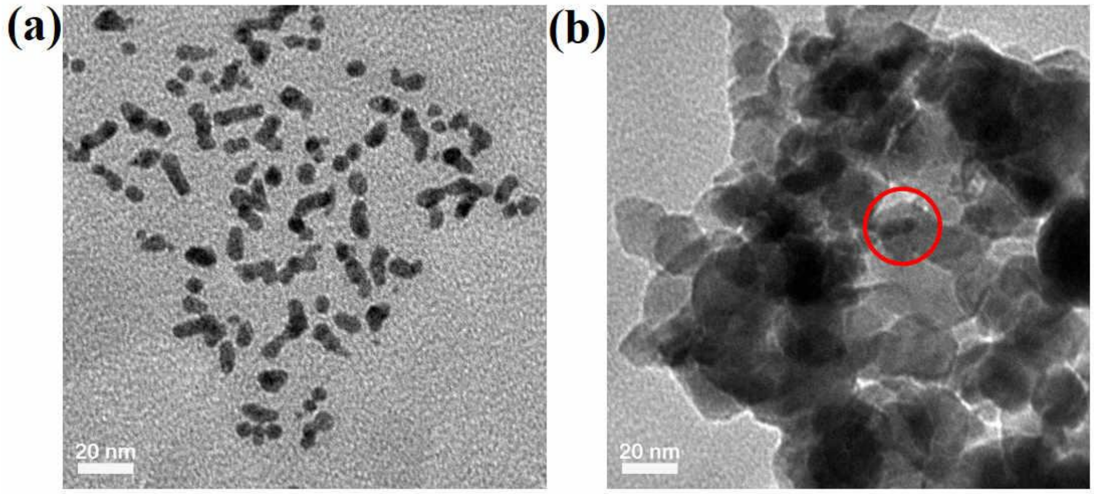

Supplementary Figure 4. | Morphology of the Pd-NP. TEM images of (a) Pd-NP nanoparticle, and (b) Pd-NP nanoparticle supported on commercial TiO_2, the Pd loading is ~4 wt.%. The scale bars are 20 nm. These TEM images show the morphologies of the nanoparticles before and after loading on TiO_2 support.

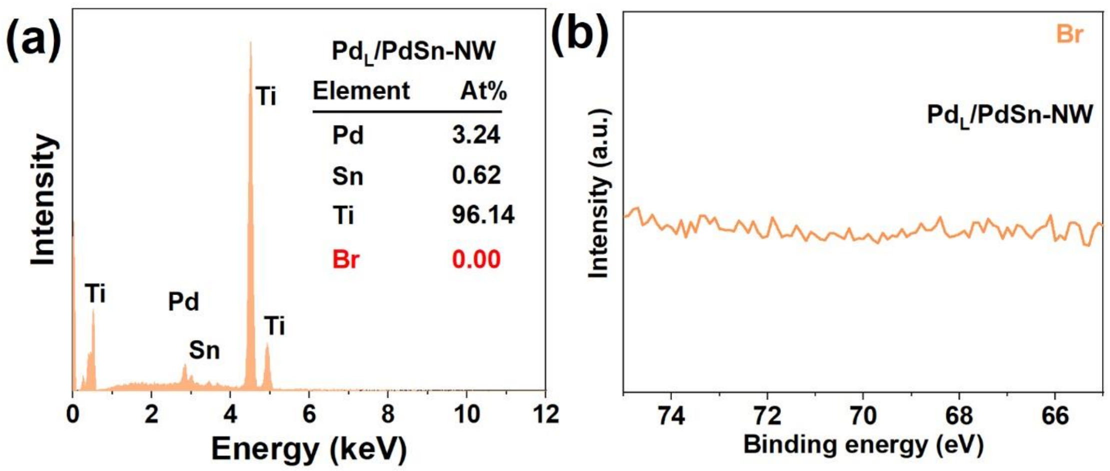

Supplementary Figure 5. | Bromine content analysis of the Pd_L/PdSn-NW. EDS (a) and XPS (b) analysis of the supported Pd_L/PdSn-NW catalyst showing the absence of Br residual.

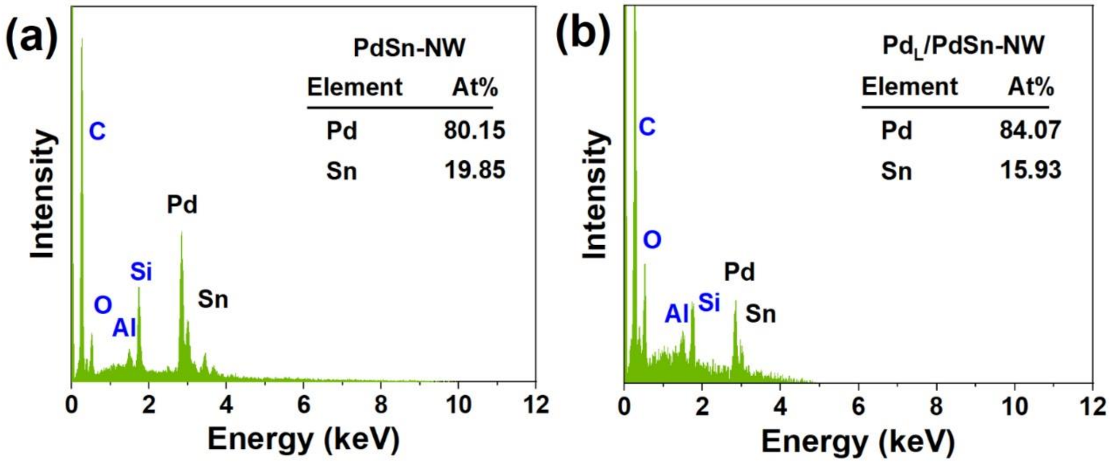

Supplementary Figure 6. | SEM-EDS characterization. SEM-EDS spectra of (a) unsupported PdSn-NW and (b) unsupported Pd$_L$/PdSn-NW showing the atomic percentages of both Pd and Sn on the two nanowires.

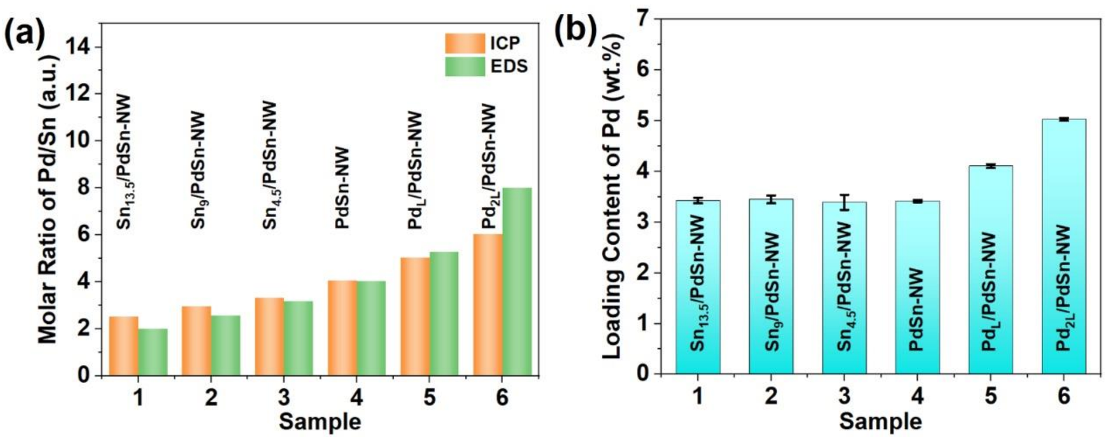

Supplementary Figure 7. | Comparison for palladium content of the catalysts. (a) Pd/Sn molar ratios of different supported samples via ICP and EDS analysis, demonstrating a good agreement between the two approaches. (b) The Pd Loadings of different supported Pd samples measured via ICP-OES analysis. The error bar shows the standard deviation. The sample names are displayed in the figures.

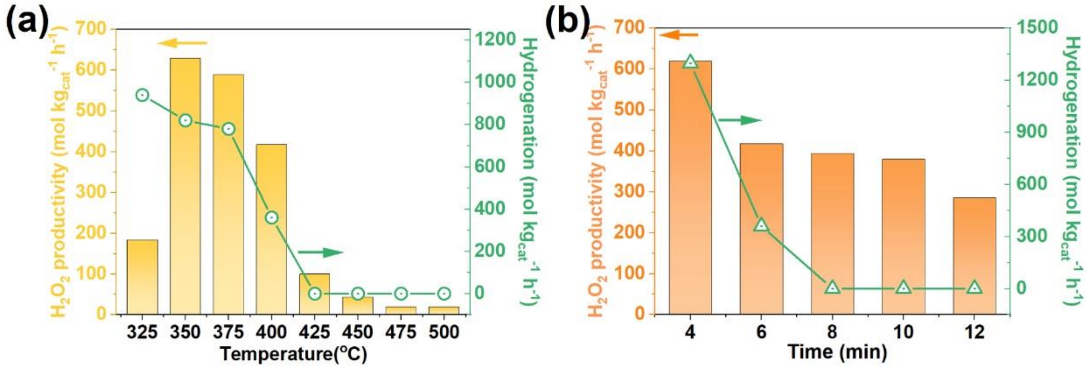

Supplementary Figure 8. | Catalytic performance of the PdSn-NW. H_2O_2 productivity and hydrogenation of the supported PdSn-NW catalyst prepared via one-step under different conditions (the support is TiO_2). (a) different annealing temperatures in air, and (b) annealing in air at 400 ºC for different times. These results indicated that the PdSn nanowire catalysts prepared via one-step is not as good as the PdSn nanowire catalyst prepared via two-step in the direct H_2O_2 synthesis.

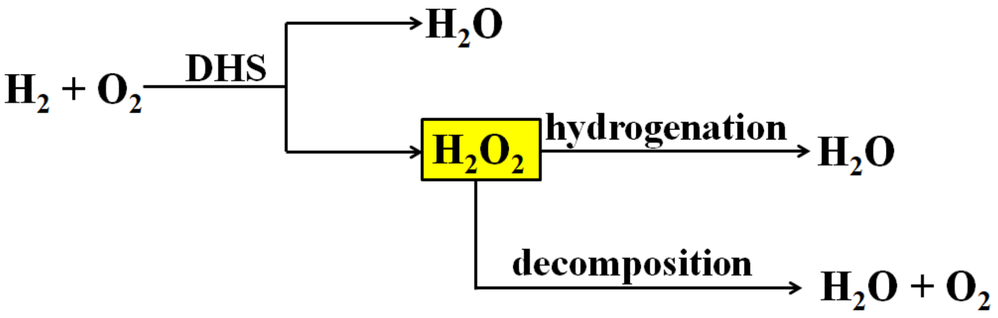

Supplementary Figure 9. | Schematic for reaction routes in the direct H_2O_2 synthesis reaction. The H_2O_2 synthesis reaction includes two processes: the direct H_2O_2 synthesis and the following dehydrogenation/decomposition. Experimental results showed that the PdSn-NW catalyst alone did not hydrogenate or decompose H_2O_2 in the second step, while the selectivity of H_2O_2 in the direct H_2O_2 synthesis from H_2 and O_2 is ~70%.

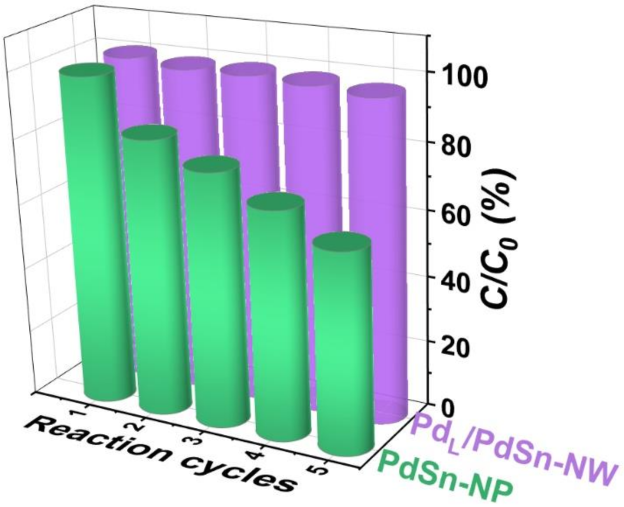

Supplementary Figure 10. | Recyclability of the Pd_L/PdSn-NW. The recyclability results of the supported Pd_L/PdSn-NW catalyst in the direct synthesis of H_2O_2, as compared to the PdSn-NP catalyst. C/C_0 is the ratio of H_2O_2 producibility produced in each run to that of the first run in the reaction cycles. The spent catalyst after each run was centrifuged and washed using ethanol/acetone mixture. After drying at 60 ºC, the material was used for the next-cycle test. During the recycle experiments, there is the catalyst loss in each run and the catalyst mass of each cycle in the experiments of recycling is shown in the table below:

**Catalyst**	**The catalyst mass of each cycle (mg)**				
	**1**	**2**	**3**	**4**	**5**
Pd_L/PdSn-NW	5.07	4.84	4.58	4.27	3.99
PdSn-NP	5.02	4.87	4.64	4.32	4.09

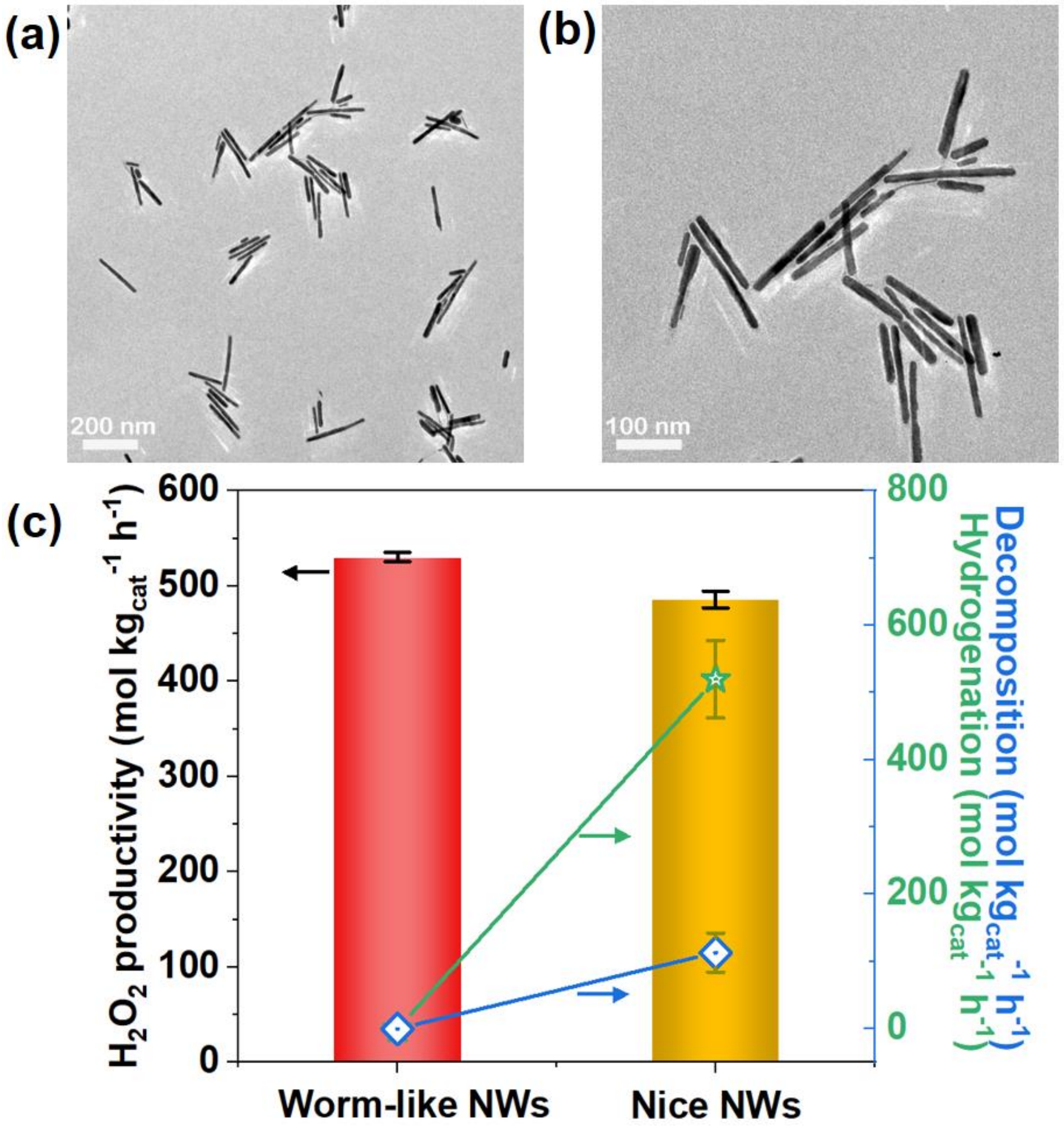

Supplementary Figure 11. | Morphology and performance of the nice PdSn nanowire. (a) and (b) TEM images of the unsupported nice PdSn nanowire prepared by the method in the literature (*Nano Lett*. 2019, 19, 6894); (c) the comparison of worm-like PdSn nanowire and the nice PdSn nanowire supported on TiO_2 in the direct H_2O_2 synthesis showing the higher decomposition/hydrogenation activity of the latter.

We have performed extra experiments by varying the experiment procedures and autoclave volumes. We have firstly performed the reaction for 15 min. Then, the reactant gas in the reactor was degassed and re-pressurized using H_2/O_2 to run the reaction for the second 15 min to achieve the H_2O_2 productivity in 30 min. The degassing and re-pressurizing were repeatedly used to achieve the H_2O_2 productivity in 45 min and 60 min, respectively. The data of the H_2O_2 productivity using this approach are shown in Supplementary Figure 12a (blue bars). As can be seen, using the above approach, there is no decrease in the H_2O_2 productivity between 15 min and 30 min runs. The H_2O_2 productivity only shows slight decrease after 30 min, as compared to the sharp decease using the conventional method by directly testing the reaction for a certain time (Supplementary Figure 12b). Therefore, the assumption of the H_2/O_2 in the autoclave over time is the one of the reasons of the decreased H_2O_2 productivity observed in the system. It is also confirmed by the tests that the H_2O_2 productivity in 60 min using a 100 mL autoclave (orange bar in Supplementary Figure 12a) is higher than that obtained in 45 min and 60 min using a 50 mL autoclave (blue bar in Supplementary Figure 12a) because the former has more H_2/O_2 molecules. Furthermore, the decrease of H_2O_2 productivity (rate) after 30 min in Supplementary Figure 11a possibly resulted from the accumulation of H_2O_2 in the autoclave according to Le Chatelier's principle or the decomposition of H_2O_2 or catalyst deactivation at high H_2O_2 concentrations.

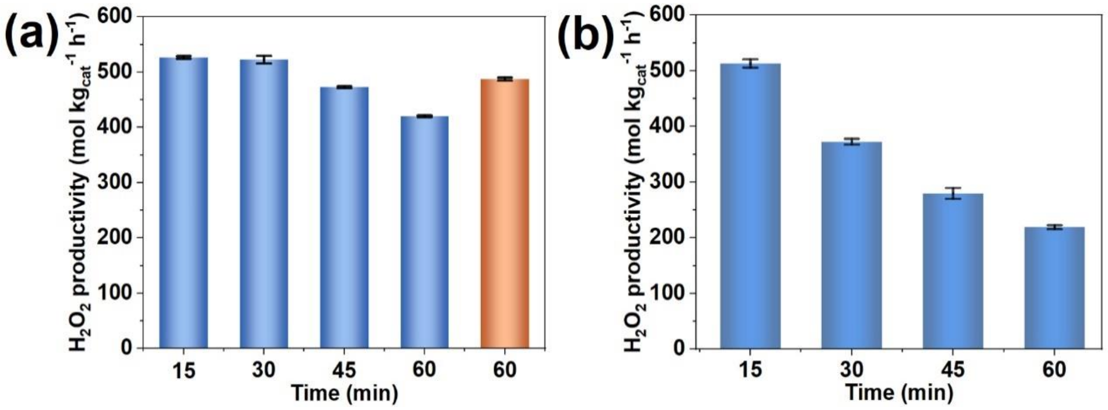

Supplementary Figure 12. | Catalytic performance of the Pd_L/PdSn-NW with time. The comparison of H_2O_2 producibility of the Pd_L/PdSn-NW catalyst in the direct H_2O_2 synthesis over time. (a) the tests were performed by repeatedly degassing and re-pressurizing H_2/O_2 at each 15 min. The data with blue color was obtained from a 50 mL autoclave and the data with orange color was obtained from a 100 mL autoclave. (b) The tests were performed by extending the reaction time in a 50 mL autoclave. The catalyst mass used in these reactions is 5 mg.

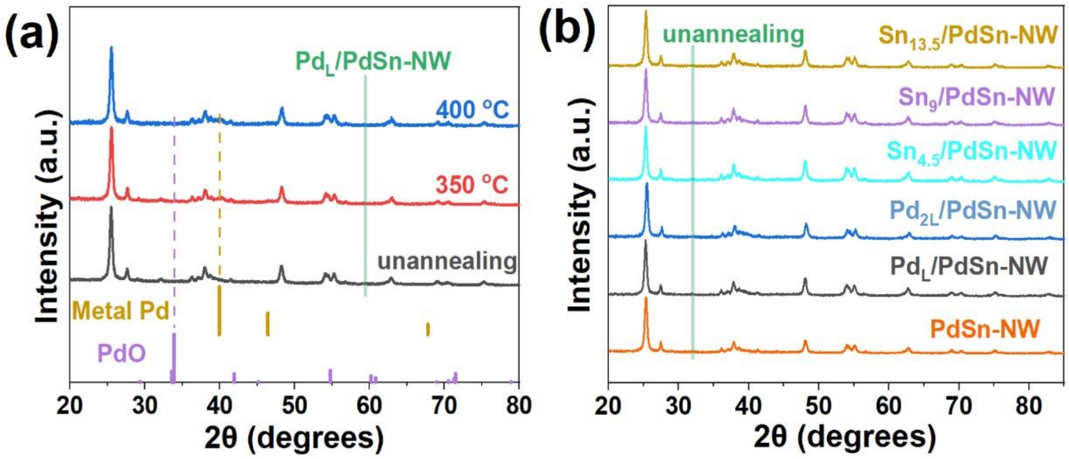

Supplementary Figure 13. | Structural characterization of the PdSn-based catalysts. XRD patterns of (a) Pd_L/PdSn-NW supported on TiO_2 annealing at different temperatures, (b) different Sn_x/PdSn-NW and Pd_x/PdSn-NW catalysts supported on TiO_2.

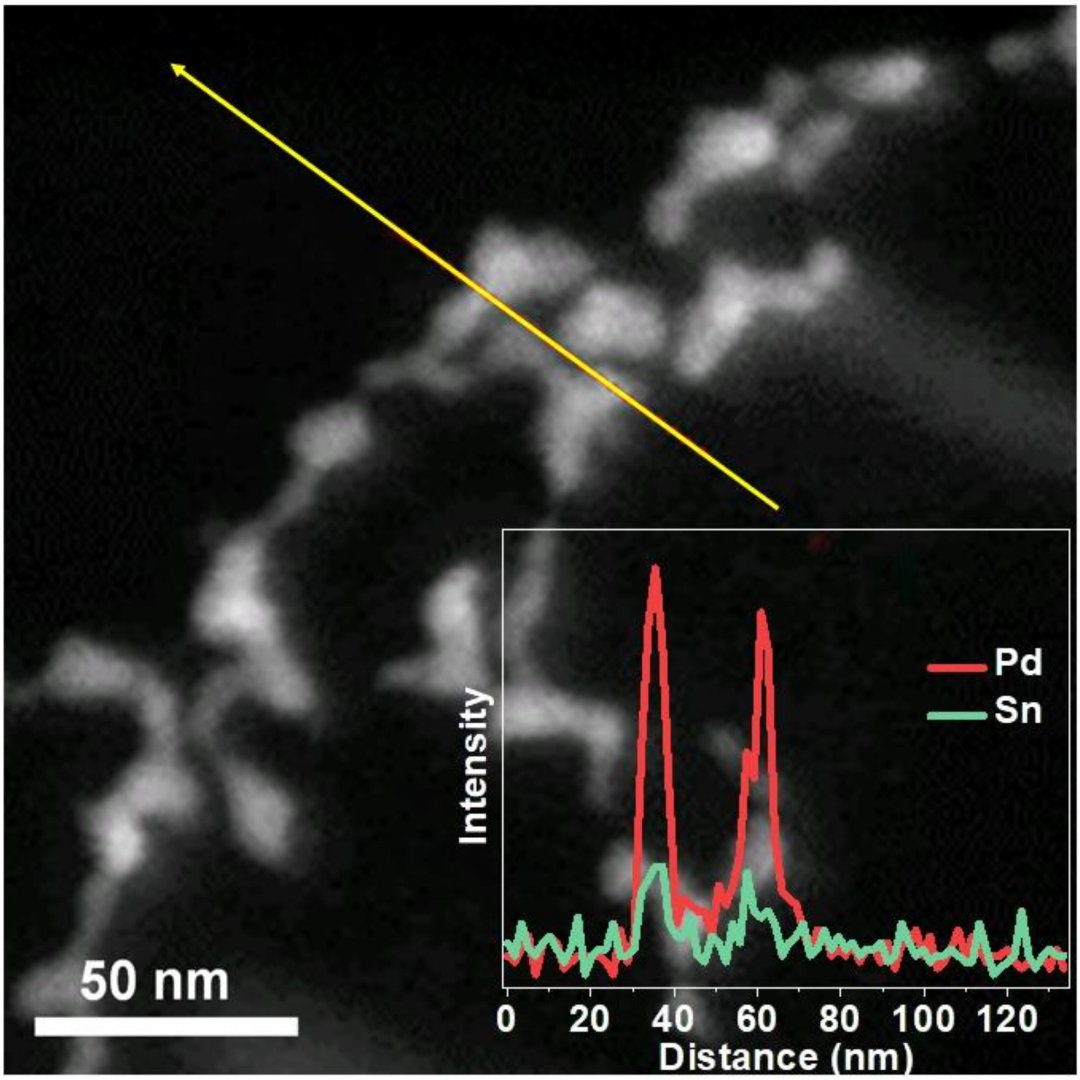

Supplementary Figure 14. | HAADF-STEM image of the Pd_L/PdSn-NW. HAADF-STEM image and line-scan analysis of the supported Pd_L/PdSn-NW catalyst annealing at 400 °C. The scale bars are 50 nm. It shows that Pd species are associated with the Sn species.

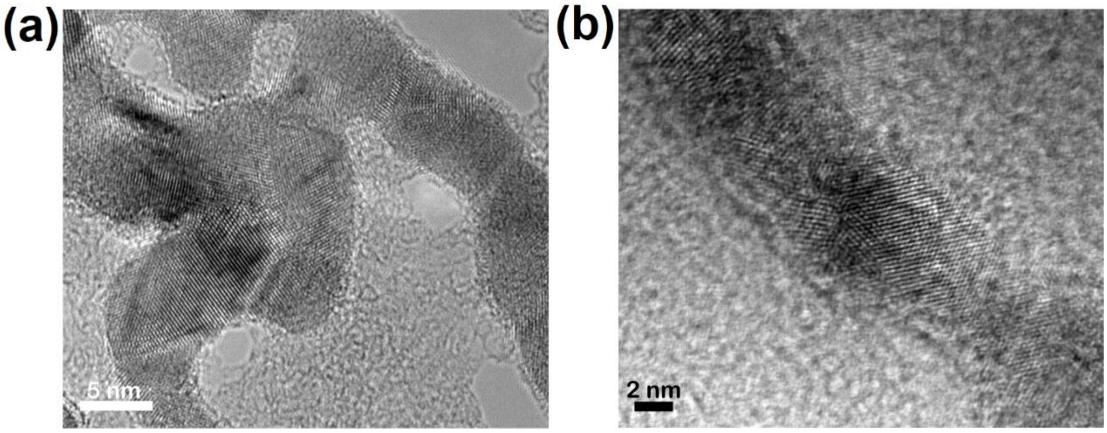

Supplementary Figure 15. | HRTEM images of the Pd_L/PdSn-NW. High-resolution transmission electron microscopy images of the supported Pd_L/PdSn-NW sample (a) after and (b) before annealing in air at 400 ºC showing the lattice fringes of the alloy. The scale bars of a and b are 5 and 2 nm, respectively.

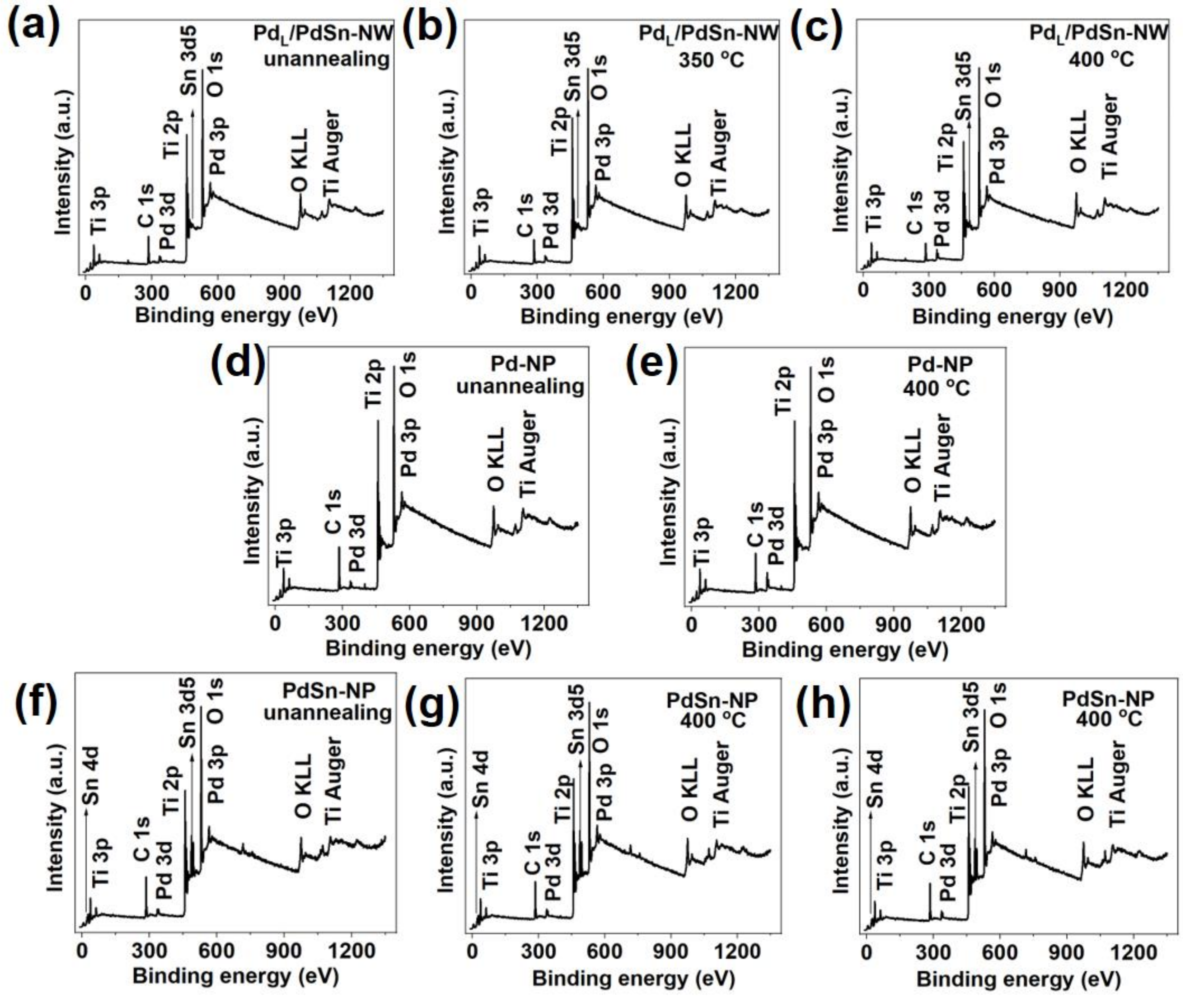

Supplementary Figure 16. | Surface speciation of the Pd-based catalysts. XPS survey spectra of different Pd samples supported on TiO_2. (a) Pd_L/PdSn-NW without annealing, (b) Pd_L/PdSn-NW annealing at 350 °C, (c) Pd_L/PdSn-NW annealing at 400 °C, (d) Pd nanoparticle catalyst without annealing, (e) Pd-NP annealing at 400 °C, (f) PdSn nanoparticle catalyst without annealing, (g) PdSn-NP sample with rapid annealing at 400 °C for 8 min and (h) PdSn-NP sample annealing at 400 °C for 4 h. All the catalysts are supported on commercial TiO_2. These XPS results indicated that no significant change was observed with the different annealing processes and XPS scanning is therefore important to determine the change of the surface species.

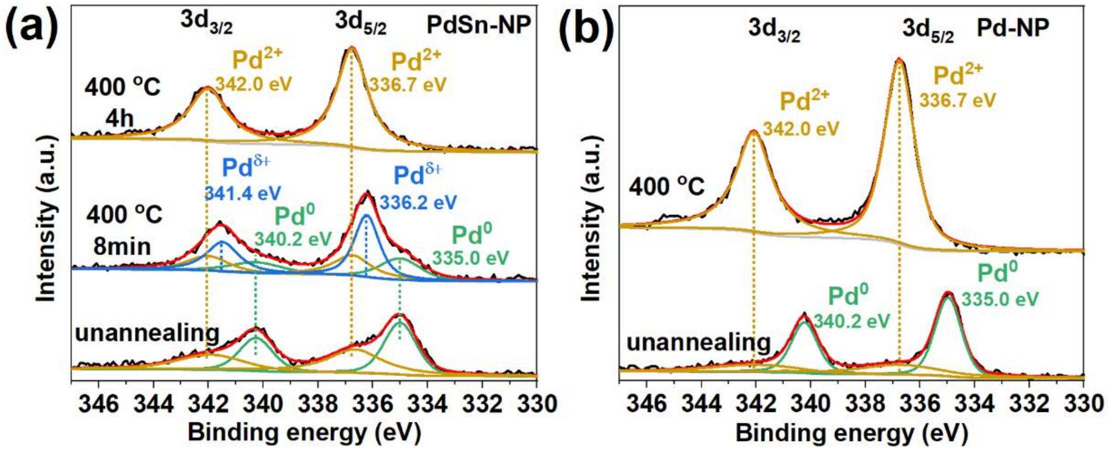

Supplementary Figure 17. | Surface speciation of the nanoparticle catalysts. XPS spectra of Pd 3*d* core level for (a) different PdSn nanoparticle samples and (b) different Pd nanoparticle samples. The catalysts are supported on commercial TiO_2.

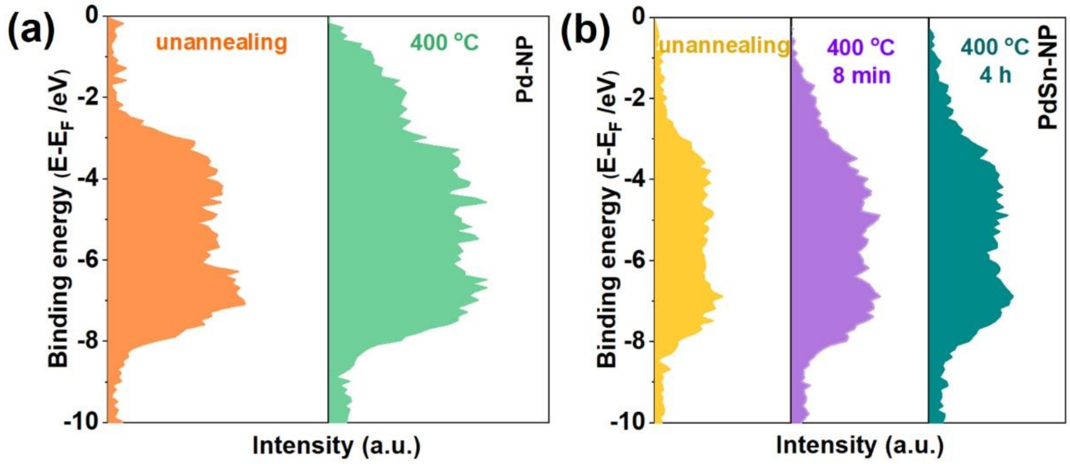

Supplementary Figure 18. | Surface characterization of catalysts. Surface valence band photoemission spectra of (a) different Pd-NP samples, and (b) different PdSn-NP samples. The catalysts are supported on commercial TiO_2.

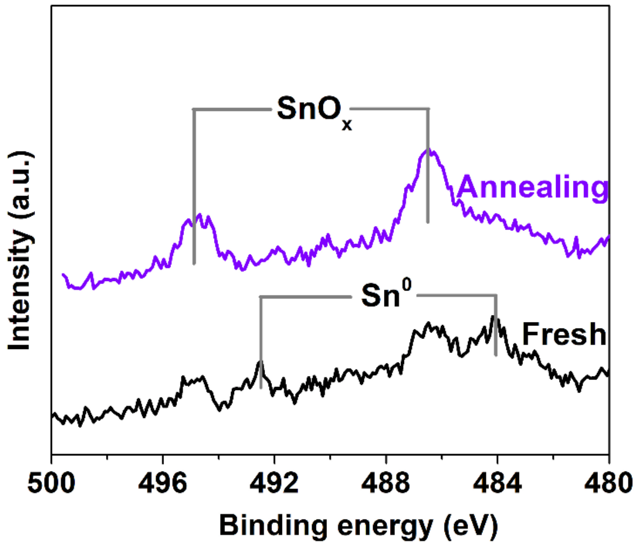

Supplementary Figure 19. | Surface tin speciation of the Pd_L/PdSn-NW. XPS spectra of Sn 3*d* of the supported Pd_L/Pd-NW catalyst before and after annealing in air.

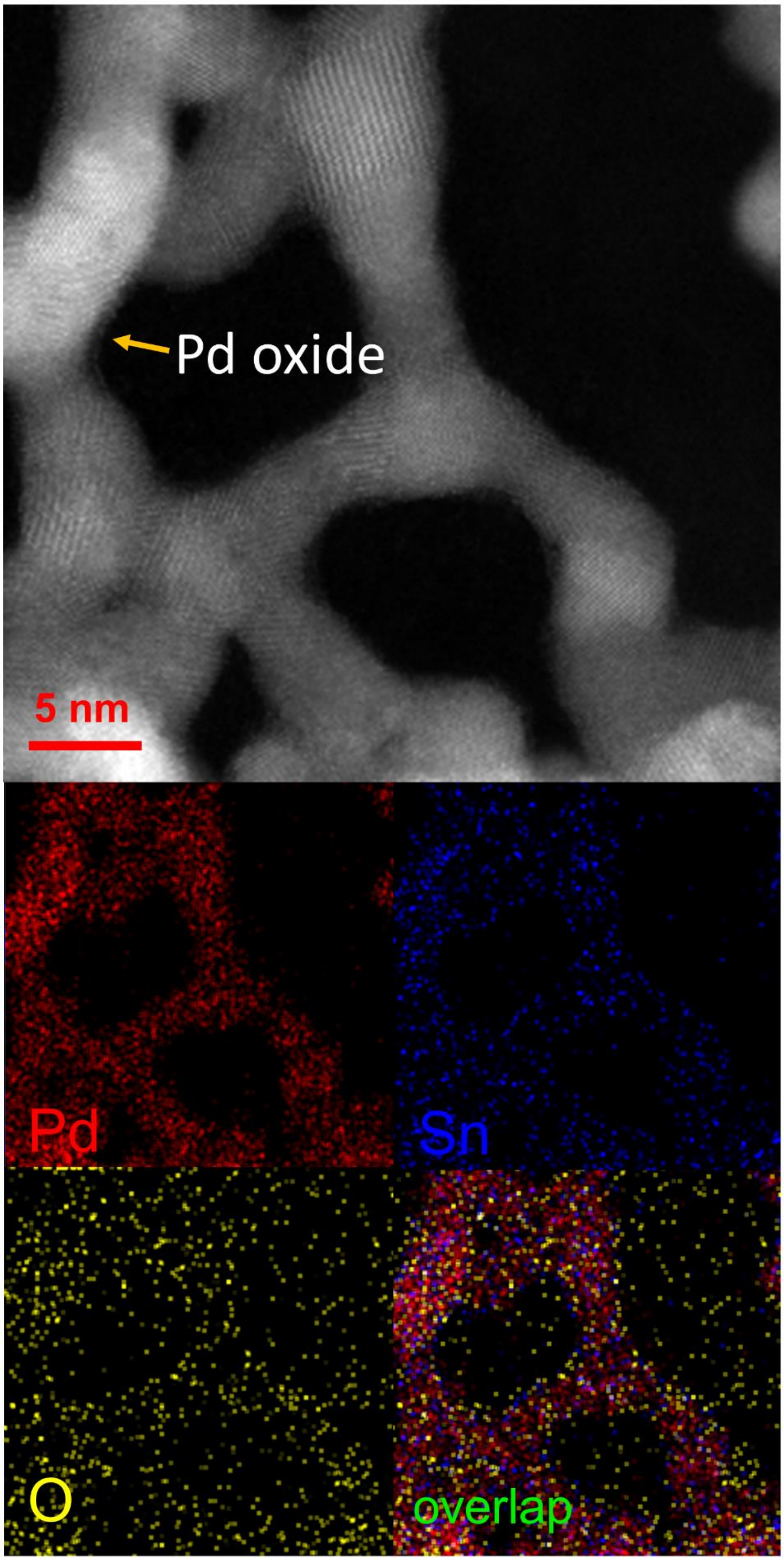

Supplementary Figure 20. | HAADF-STEM image and elemental mapping of Pd_L/PdSn-NW catalyst supported on TiO_2. The scale bars are 5 nm.

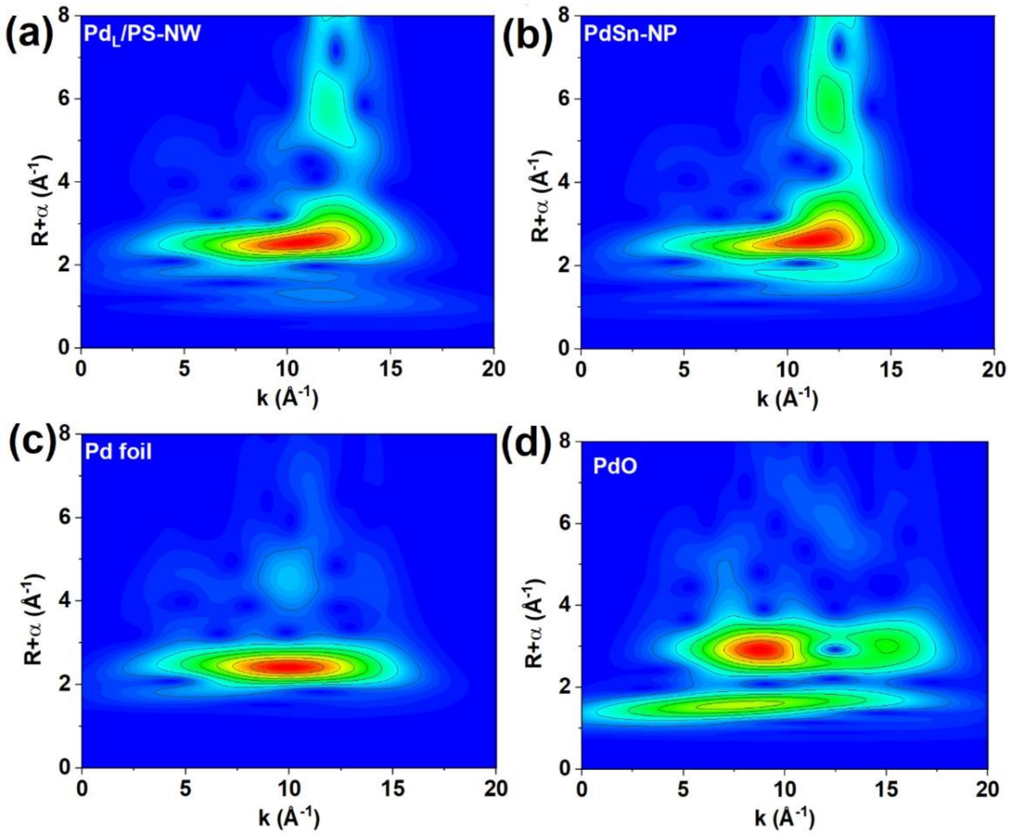

Supplementary Figure 21. | Wavelet transform for EXAFS spectra of PdSn catalysts and the references. (a) supported Pd_L/PdSn-NW sample annealing at 400 ºC, (b) supported PdSn-NP sample annealing at 400 ºC for 8 min, (c) reference Pd foil and (d) reference PdO sample.

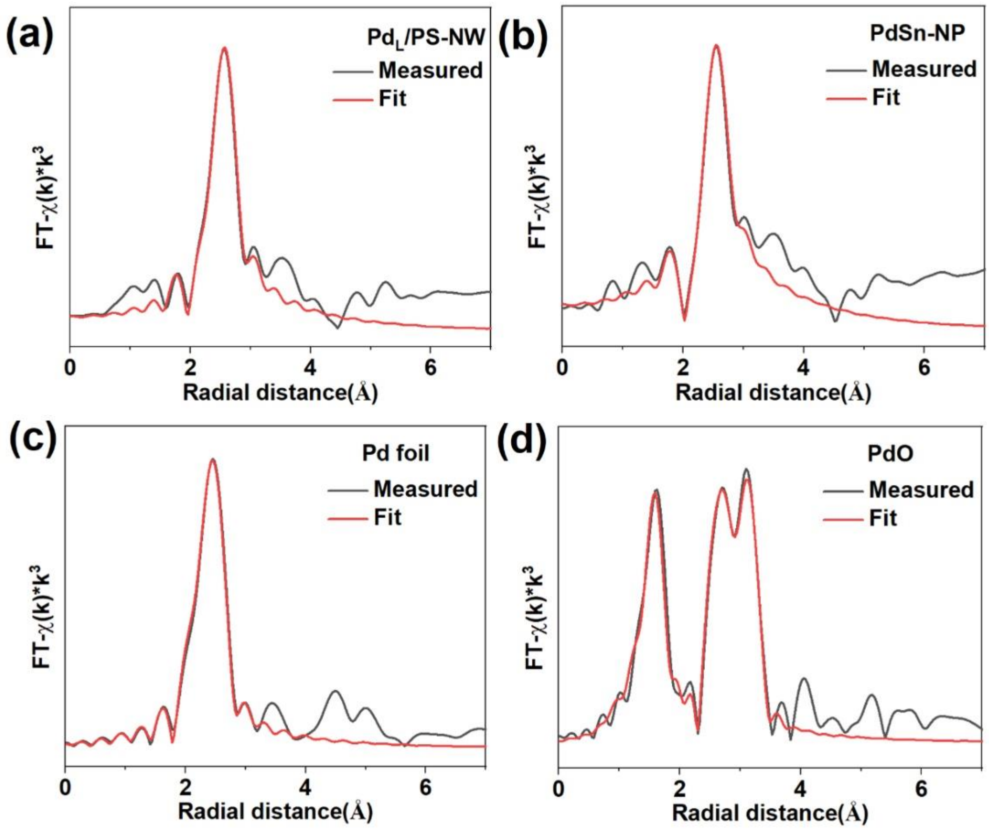

Supplementary Figure 22. | EXAFS fitting curves of catalysts. Pd K-edge EXAFS fitting curves at R space for supported PdSn nanowire catalysts and the reference samples. (a) Pd_L/PdSn-NW annealing at 400 ºC, (b) PdSn-NP annealing at 400 ºC for 8 min, (c) reference Pd foil, and (d) reference PdO. The catalyst support is TiO_2.

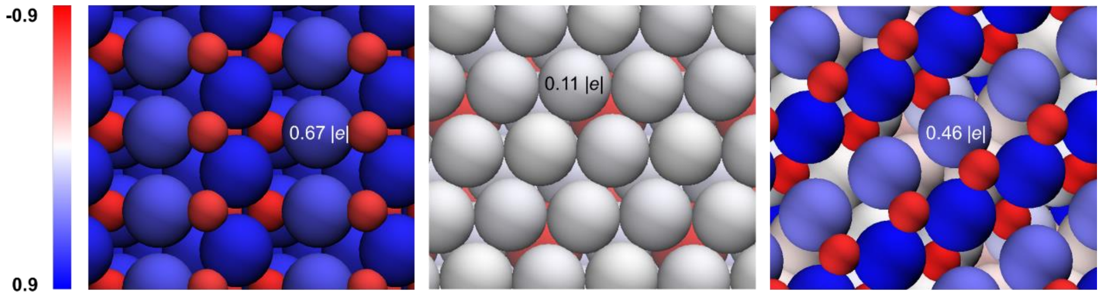

Supplementary Figure 23. | Bader charge of surface Pd. Charge state distribution on PdO(101), Pd_4Sn and PdO@Pd_4Sn with atomic coloring according to their Bader charges.

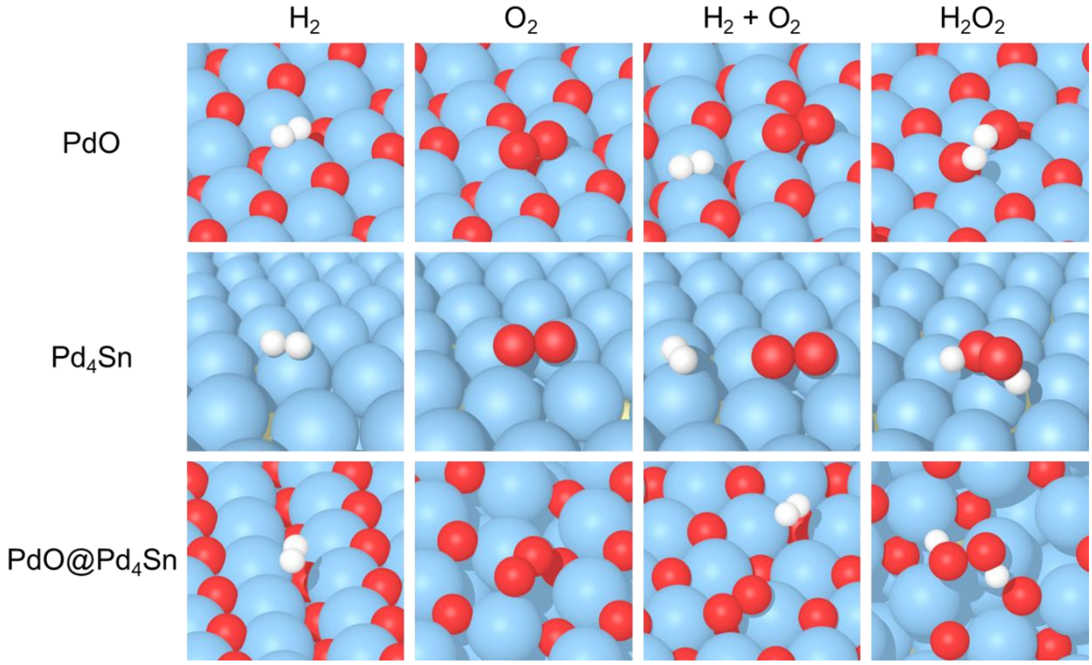

Supplementary Figure 24. | Optimized structures for the adsorptions of key intermediates. Configurations of H_2, O_2, H_2+O_2 and H_2O_2 adsorption on the PdO, Pd_4Sn and PdO@Pd_4Sn surfaces. Color code: blue, Pd; red, O; white, H; yellow, Sn.

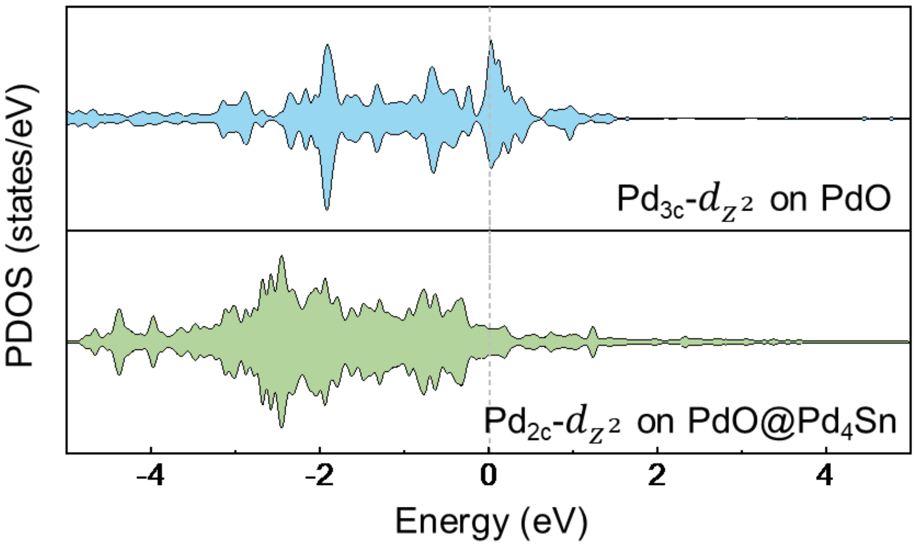

Supplementary Figure 25. | Calculated projected density of states. Projected density of state (PDOSs) of surface Pd_{3c}-d_{z^2} on PdO and Pd_{2c}-d_{z^2} on PdO@Pd_4Sn, Fermi level was set to 0 eV.

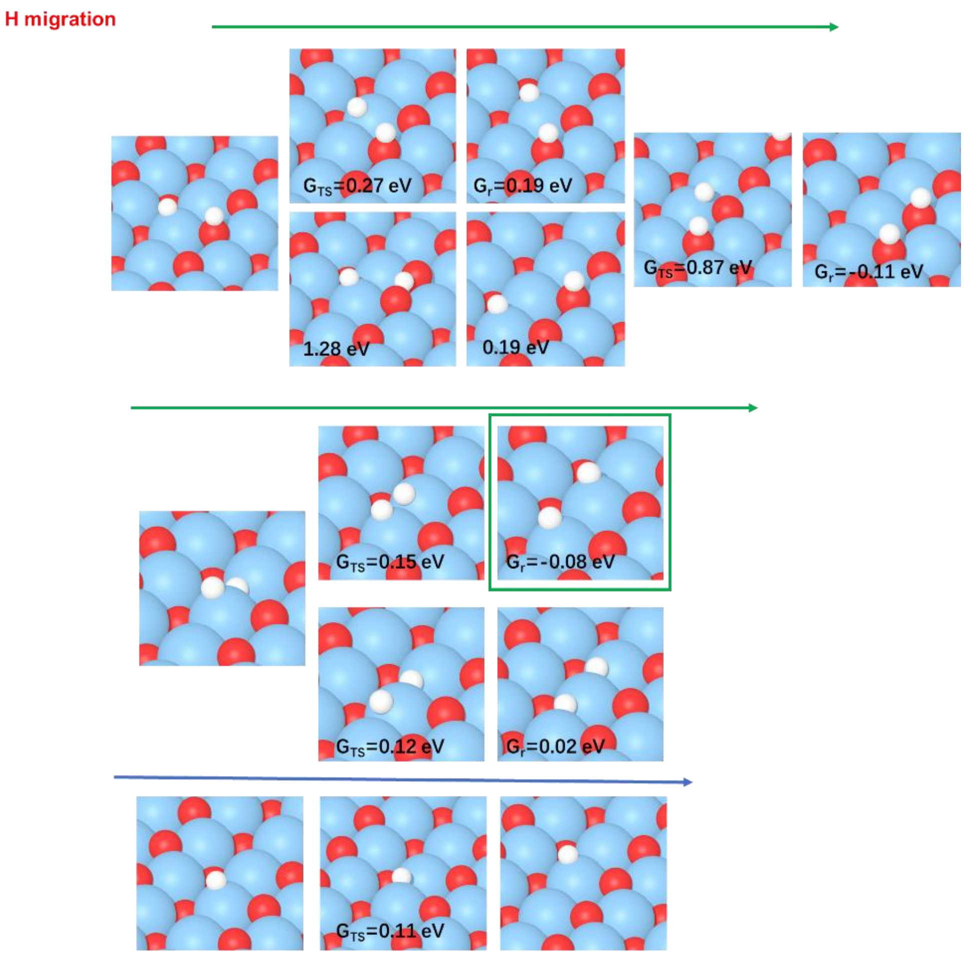

Supplementary Figure 26. | Processes of hydrogen migration on PdO(101). H migrations via various pathways on PdO(101), starting from the H_2 dissociation products.

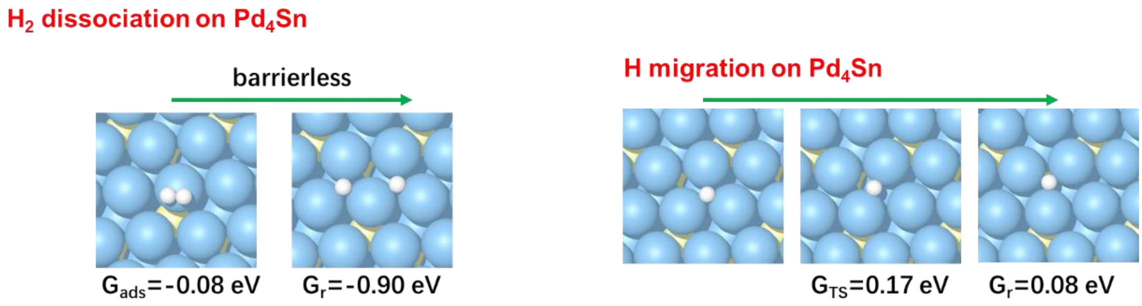

Supplementary Figure 27. | Spontaneous H_2 dissociation on Pd_4Sn. IS and FS of H_2 dissociation above Pd_4Sn (left), H migration above Pd_4Sn (right).

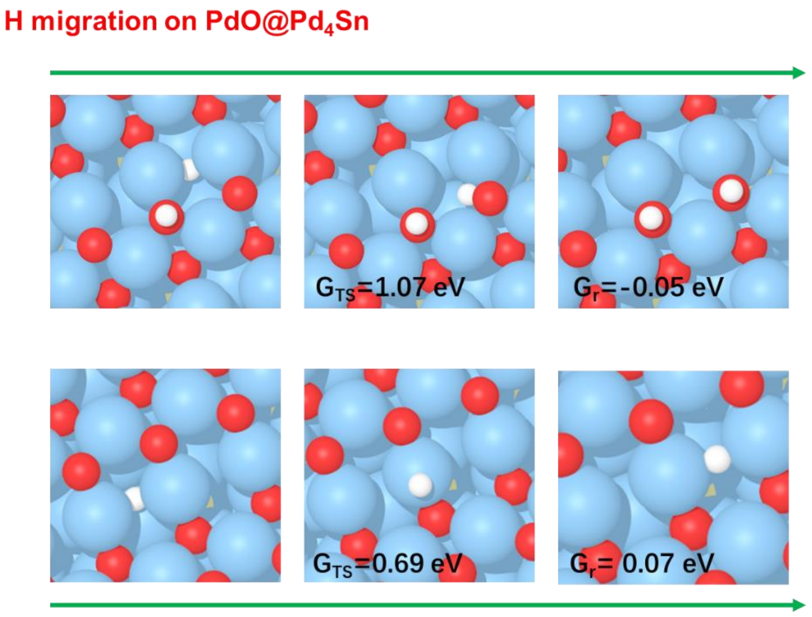

Supplementary Figure 28. | Processes of hydrogen migration on PdO@Pd_4Sn. H migrations via various pathways on PdO@Pd_4Sn. Since H is not stable on the Pd top site and it prefers to locate at the bridge site, thus the conversion between top and bridge sites was not considered.

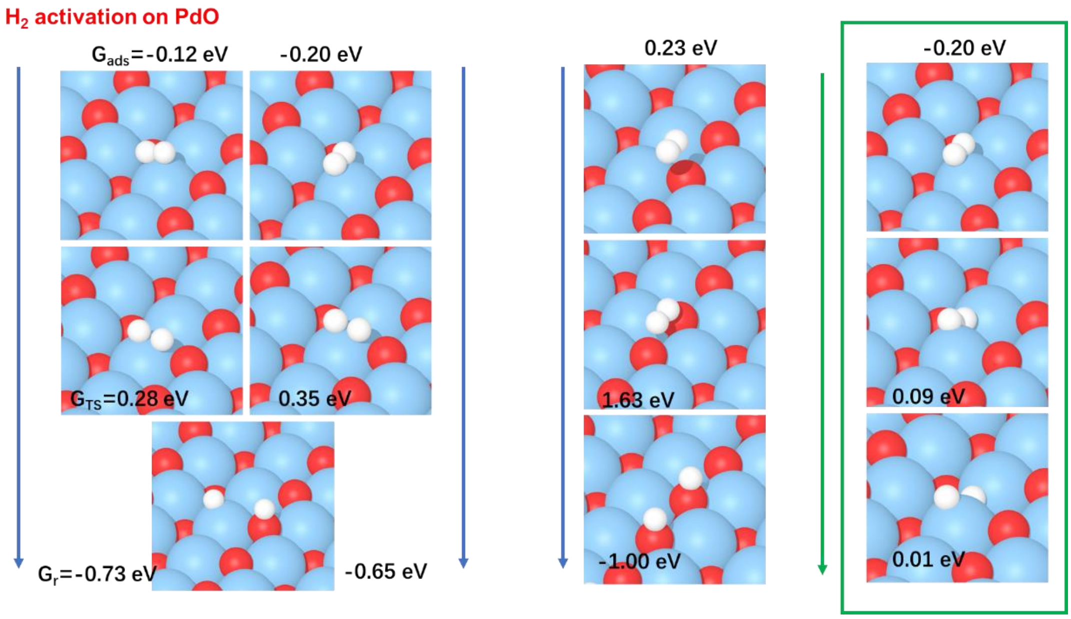

Supplementary Figure 29. | Processes of H_2 dissociation on PdO(101). Various pathways of H_2 activation on PdO(101), the path highlighted by green frame is the optimum path that demonstrated in main text. E_{ads}, E_{TS} and E_r represent the H_2 adsorption free energy, free energy barrier and reaction free energy, respectively.

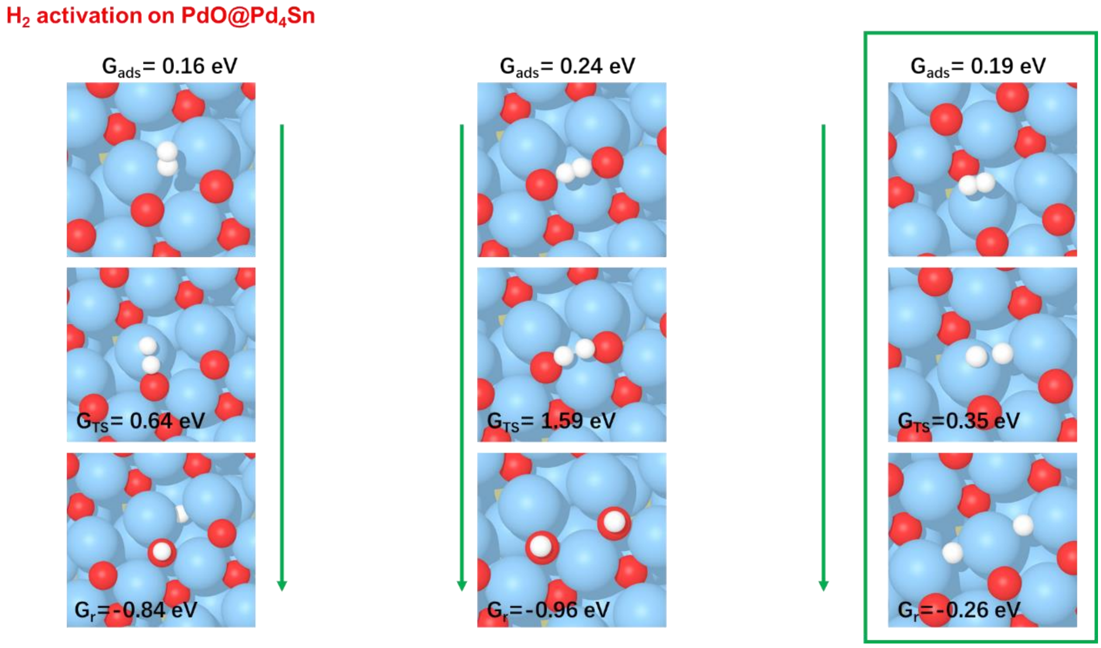

Supplementary Figure 30. | Processes of H_2 dissociation on PdO@Pd_4Sn. Various pathways of H_2 activation on PdO@Pd_4Sn.

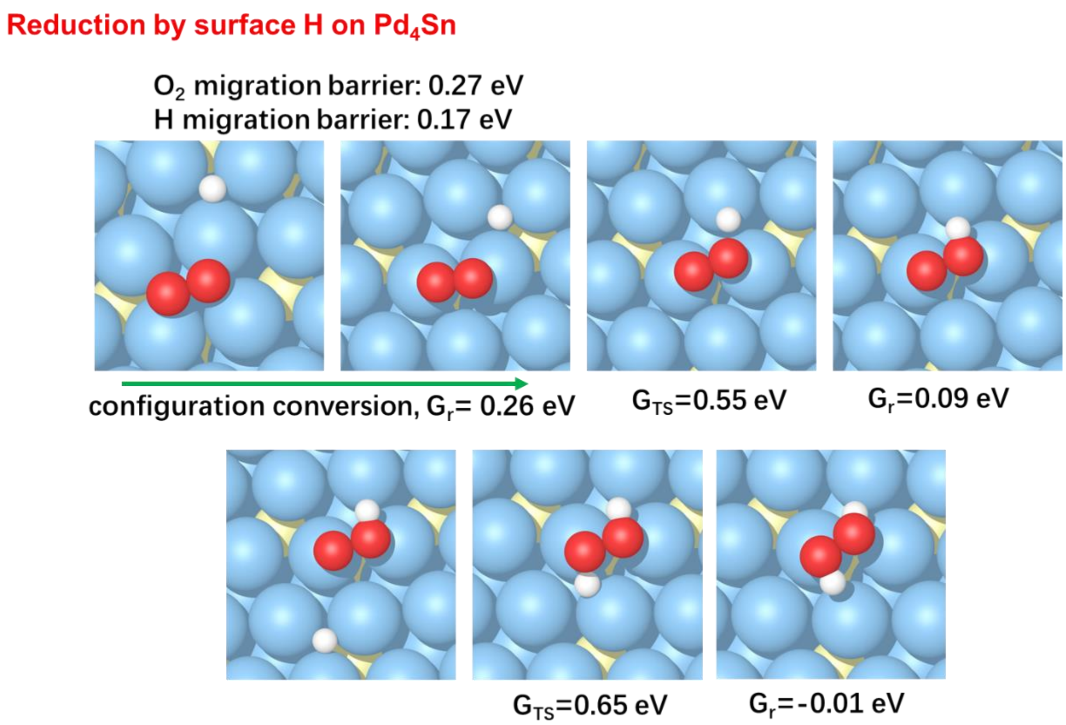

Supplementary Figure 31. | Processes of O_2 reduction on Pd_4Sn. Configurations of the intermediates during O_2 reduction by Pd-H on Pd_4Sn.

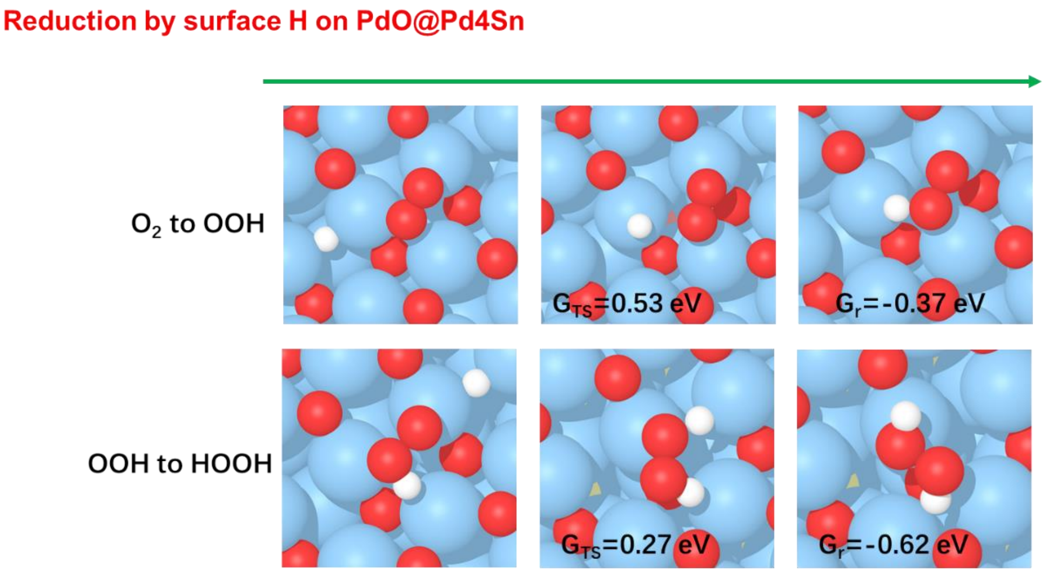

Supplementary Figure 32. | Processes of O_2 reduction on PdO@Pd_4Sn. Configurations of the intermediates during O_2 reduction by Pd-H on PdO@Pd_4Sn.

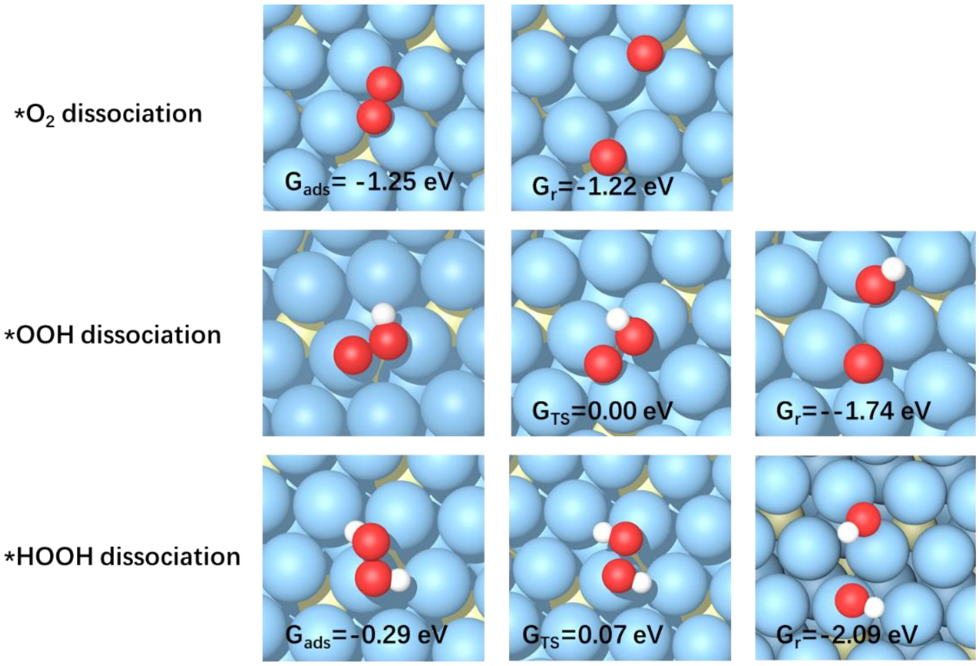

Supplementary Figure 33. | Side reactions on $Pd_4Sn(111)$. Structures of IS, TS and FS of $*O_2$, *OOH and *HOOH dissociation catalyzed by $Pd_4Sn(111)$, with free energy barriers (G_{TS}), reaction free energies (G_r) and adsorption free energies (G_{ads}).

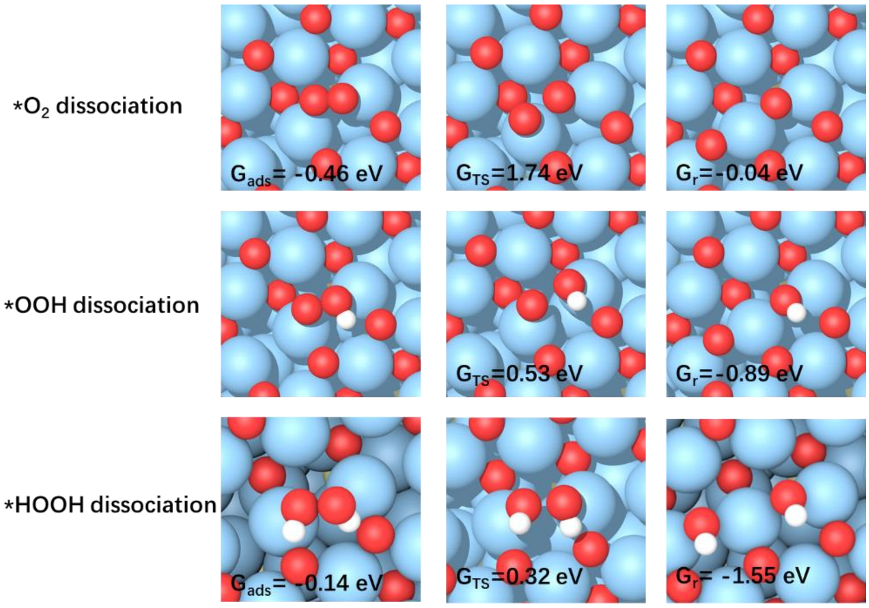

Supplementary Figure 34. | Side reactions on PdO@Pd_4Sn. Structures of IS, TS and FS of *O_2, *OOH and *HOOH dissociation catalyzed by PdO@Pd_4Sn, with free energy barriers (G_{TS}), reaction free energies (G_r) and adsorption free energies (G_{ads}).

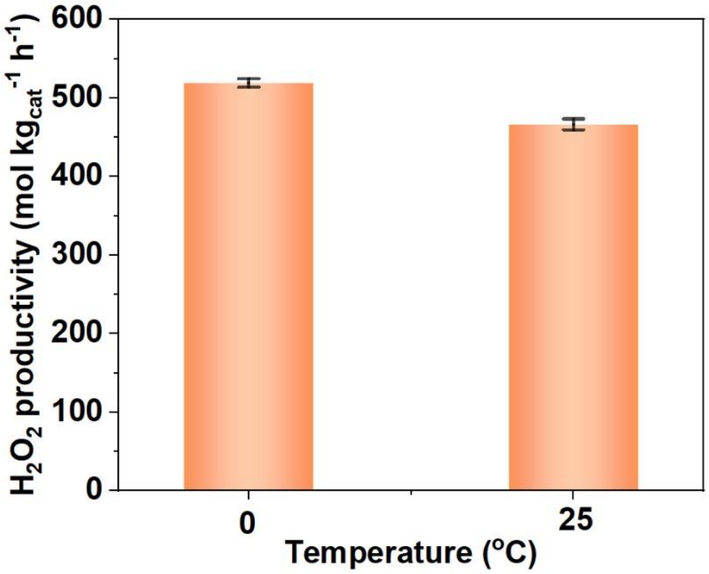

Supplementary Figure 35. | Catalytic performance of the Pd$_L$/PdSn-NW with temperature. The H_2O_2 producibility comparison of the supported Pd$_L$/PdSn-NW catalyst carried out at 0 Celsius and 25 Celsius.

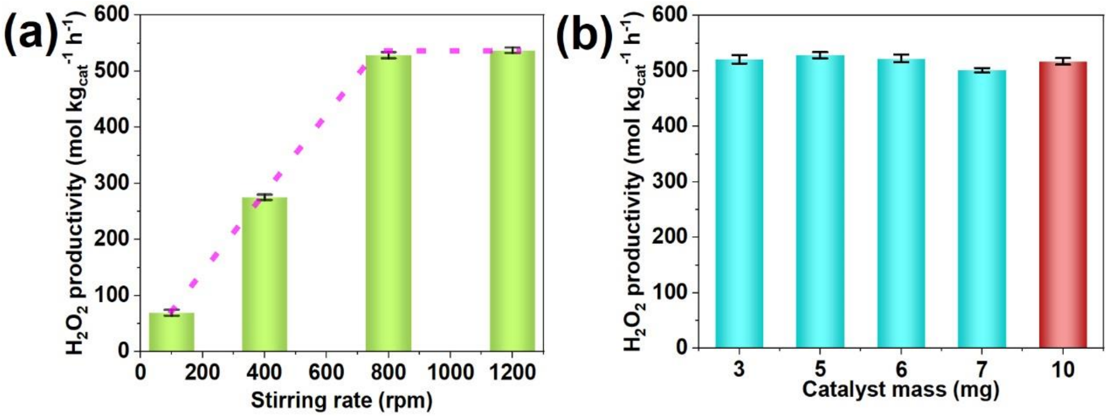

Supplementary Figure 36. | Comparison for catalytic performance of the Pd$_L$/PdSn-NW with different conditions. The comparison of H_2O_2 producibility of the supported Pd$_L$/PdSn-NW catalyst in the direct H_2O_2 synthesis under (a) different stirring rate and (b) varying catalyst mass indicating there is no interphase mass transfer constraints under the reaction conditions reported. The data in a were obtained using a 50 mL autoclave. The data with the light blue in b were carried out in a 50 mL autoclave and the data with the red color in b was obtained from a 100 mL autoclave.

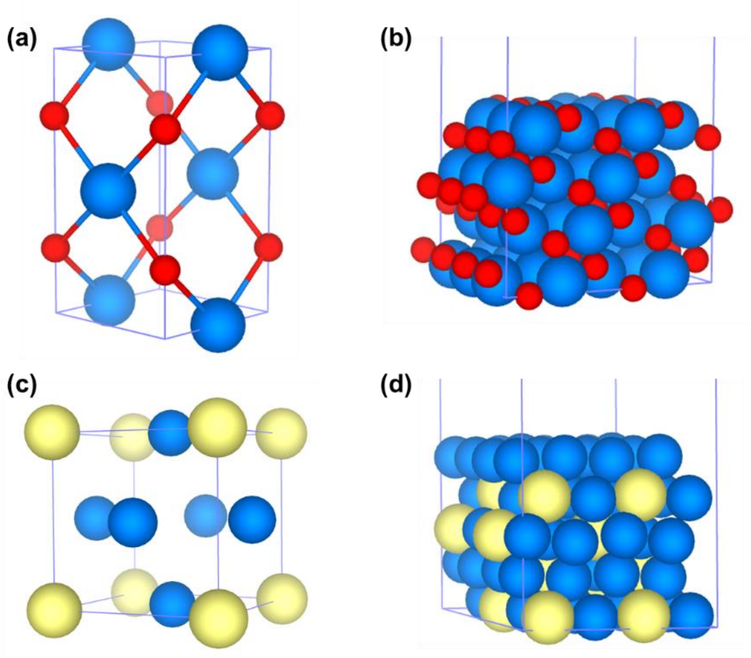

Supplementary Figure 37. | Computational Models. Models of (a) PdO unitcell, (b) PdO (101), (c) Pd_3Sn and (d) Pd_4Sn (111). Color code: red, O; blue, Pd; yellow, Sn.

Supplementary Notes

Supplementary Note 1. Calculation of the thickness of Pd layer on PdSn nanowires prepared by the two-step solvothermal approach.

For the estimation, the PdSn nanowires prepared by the first step were approximately considered as the cylinder model, and the Pd precursor which was further added were assumed as the sphere model.

Precursor addition amount: $m_1[Pd(acac)_2]$=7.6 mg, $m_2[Pd(acac)_2]$=1.9 mg

The number of palladium atoms:

$$N_{1Pd} = \frac{7.6\varphi}{1000\times304.6} \times N_A, \quad (1)$$

$$N_{2Pd} = \frac{1.9\varphi}{1000\times304.6} \times N_A \quad (2)$$

φ is the yield of the nanowires material obtained during the synthetic process.

$$V_{1total} = V_{1Pd} \times N_{1Pd} \quad (3)$$

$$Q_{NWs} = V_{1total} \,/\, (\tfrac{4}{5} \times V_{NWs}) \quad (4)$$

$$S_{2total} = S_{2Pd} \times N_{2Pd} \quad (5)$$

Considering that the length is much larger than the diameter of the nanowires, we think the lateral area of nanowires can approximately regarded as the surface area.

$$S_{NWs} = 2\pi R_{NWs}h + 2 \times \pi R_{NWs}^2 \approx 2\pi R_{NWs}h \quad (6)$$

$$\begin{aligned} L &= S_{2total} \,/\, (S_{NWs} \times Q_{NWs}) \\ &\approx (S_{2Pd} \times N_{2Pd}) \times (\tfrac{4}{5} \times V_{NWs}) \,/\, (2\pi R_{NWs}h \times V_{1total}) \\ &= (S_{2Pd} \times N_{2Pd}) \times (\tfrac{4}{5} \times V_{NWs}) \,/\, (2\pi R_{NWs}h \times \tfrac{4}{3} \times \pi \times r_{Pd}^3 \times \tfrac{7.6\varphi}{1000\times304.6} \times N_A) \\ &= (\pi r_{Pd}^2 \times \tfrac{1.9\varphi}{1000\times304.6} \times N_A) \times (\tfrac{4}{5} \times \pi \times \tfrac{D_{NWs}^2}{4} \times h) \,/\, (\pi D_{NWs}h \times \tfrac{4}{3} \times \pi \times r_{Pd}^3 \tfrac{7.6\varphi}{1000\times304.6} \times N_A) \\ &= 1.9 \times \tfrac{D_{NWs}}{5} \,/\, (\tfrac{4}{3} \times r_{Pd} \times 7.6) \\ &= 3 \times 1.9 \times D_{NWs} \,/\, (20 \times r_{Pd} \times 7.6) \\ &= 1.15 \end{aligned} \quad (7)$$

Therefore, the average number of layers covered by Pd atoms on the pre-prepared PdSn nanowire is 1.15 layers and the PdSn nanowire catalyst prepared via two-step approach is denoted as Pd_L/PdSn-NW.

Supplementary Tables

Supplementary Table 1. Comparison of catalytic reactivity of PdSn nanowire catalyst in this work and the well-developed catalysts in literature for the direct H_2O_2 synthesis

Catalyst	Pd loading (wt.%)[c]	Synthesis Conditions	H_2O_2 Prod. (mol/kg_{cat}*h) [d]	H_2O_2 Prod. (mol/kg_{pd}*h)	Hydrog/Decomp Conditions	H_2O_2 hydrogenation (mol/kg_{cat}*h)	H_2O_2 decomposition (mol/kg_{cat}*h)	Ref.
Pd_L/PdSn-NW	4.1 (0.9)	4.0 MPa (5% H_2, 10% O_2, 85% Ar), 0 °C, 5 mg cat., 50 mL autoclave	528 (373)	12878	3.6 MPa (5% H_2, 95% Ar) / 3.6 MPa N_2, 0 °C, 5 mg cat.	0	0	This work
Pd_L/PdSn-NW[a]	4.1 (0.9)		630	18529		820	360	This work
Pd_6Pb NRs/TiO_2	3.1 (1.1)	4.0 MPa (5% H_2, 10% O_2, 85% Ar), 0 °C, 5 mg cat., 60 mL autoclave	(170.1)	5667	3.6 MPa (5% H_2, 95% Ar) / 3.6 MPa N_2, 0 °C, 5 mg cat.	~260	~0.9	*ACS Catalysis* 2021, **11**, 1106
AuPd@HZSM-5	2.4 (2.3)	4.0 MPa (5% H_2, 10% O_2, 45% Ar, 40% He), 0 ± 2 °C, 30 mg cat., 30 mL autoclave	(320)	12800	No test /2 MPa (20%O_2, 80% He), 0 ± 2 °C, 30 mg cat.	/	41	*ACS Catalysis* 2021, **11**, 1946
Pd_3Pb/s-TiO_2	2.4 (1.7)	4.0 MPa (4% H_2, 20% O_2, 76% N_2), 30 °C, ca. 32 mg cat., 300 mL autoclave	(176.1)	7339	/	/	/	*ACS Catalysis* 2021, **11**, 2288
1% Pd NP/C	1.3 (/)	4.0 MPa (5% H_2, 25% O_2, 70% CO_2), 2 °C, 10 mg cat., 100 mL autoclave	(120)	12000	2.9 MPa (5% H_2, 95% CO_2)/ No test , 2 °C, 10 mg cat.	360	/	*ACS Catalysis* 2020, **10**, 5928
R–PdNi/TiO_2-C[b]	0.85 (0.42)	0.1 MPa (2.5% H_2, 50% O_2, 47.5% Ar), 20 °C, 5 mg cat., 100 mL reactor	(96.6)	11364	/	/	/	*ACS Catalysis* 2021, **11**, 8407
3 wt % Pd-2 wt % Sn/TiO_2	3 (2)	4.0 MPa (5% H_2, 25% O_2, 70% CO_2), 2 °C, 10 mg cat., 100 mL autoclave	(61)	2033	2.9 MPa (5% H_2, 95% CO_2) /0.1 MPa (air) , 2 °C, 10 mg cat.	0	/	*Science* 2016, **351**, 965
2.5% Au-2.5% Pd/carbon	2.5 (2.5)	4.0 MPa (5% H_2, 25% O_2, 70% CO_2), 2 °C, 10 mg cat., 100 mL autoclave	(160)	6400	2.9 MPa (5% H_2, 95% CO_2) /0.1 MPa (air) , 2 °C, 10 mg cat.	0	0	*Science* 2009, **323**, 1037

[a] Annealing temperature is 350 °C

[b] The catalytic reaction solvent contains 50 mL D_2O and 0.5 M sulfuric acid

[c] The parenthesis is the loading of the second metal in the Pd-M alloy catalysts

[d] The parenthesis is the producibility of the catalysts performed in 30 min.

Supplementary Table 2 The actual amount of H_2O_2 producibility of the Pd catalysts as ppm and wt.% in the direct H_2O_2 synthesis from H_2 and O_2 at zero Celsius.

Catalyst	H_2O_2 productivity (mol/kg*h)	Reaction time (min)	Concentration (ppm)	Concentration (wt.%)
Pd_L/PdSn-NW	528	15	2244	0.26
Pd_L/PdSn-NW	373	30	3171	0.37
PdSn-NW	398	15	1692	0.20
PdSn-NP	99	15	420	0.05
Pd-NP	68	15	289	0.03
3 wt.%Pd-2 wt.%Sn/TiO_2*	61	30	1037	0.21

*: the catalyst was reported in the reference of *Science* 2016, 351, 965

Supplementary Table 3 Fit parameters of Pd K-edge EXAFS for PdSn catalysts and references.

Samples	Path	N [a]	R/Å [b]	σ^2/Å^{-2} [c]	ΔE/eV [d]	R-factor [e](%)
Pd_L/PdSn-NW	Pd-Pd	4.2 (0.3)	2.76 (0.01)	0.003 (0.001)	0.91 (0.5)	1.6
	Pd-Sn	0.8 (0.1)	2.57 (0.02)	0.003 (0.001)		
PdSn-NP	Pd-Pd	3.3 (0.2)	2.74 (0.01)	0.003 (0.002)	-2.45 (0.6)	2.3
	Pd-Sn	0.13 (0.1)	2.53 (0.02)	0.01 (0.001)		
Pd foil	Pd-Pd	12 (0.4)	2.74 (0.02)	0.005 (0.001)	-7.71 (1.4)	1.4
PdO	Pd-Pd	4 (0.4)	3.04 (0.03)	0.005 (0.001)	-4.31 (1.2)	1.5
	Pd-O	4 (0.3)	2.00 (0.01)	0.002 (0.001)	-4.31 (1.2)	

[a] Coordination number
[b] Interatomic distance
[c] Debye-Waller factor
[d] Shift of the energy threshold
[e] R factor indicating the goodness of fit between experimental and theoretical data

The measurements were conducted in the transmission mode in TLS-01C beamline of the National Synchrotron Radiation Research Center (NSRRC, Hsinchu, Taiwan). EXAFS data was processed and fitted with the ARTEMIS software package. The powders were pressed into pellets for XAS measurements. The energy calibration of the catalysts was conducted using Pd foil and PdO as standard references, which were simultaneously measured. For EXAFS data, the EXAFS of Pd foil was fitted by determining the coordination number (CN) of Pd-Pd bond as 12 and the obtained amplitude reduction factor S_0^2 value (0.9) was set in the EXAFS analysis to determine the CN in the Pd-Pd and Pd-Sn scattering path in all catalysts. ΔE_0 was used for all the shells, while σ^2 and ΔR values were used for each shell for the same type of back-scattering atoms. EXAFS fittings were conducted to investigate the 1st shell coordination structures of the Pd atoms on the catalysts.
