# Supplementary material for: Layered Pd oxide on PdSn nanowires for boosting direct H2O2 synthesis
Source: Nat Commun. 2022 Oct 14;13:6072. doi: 10.1038/s41467-022-33757-0 (PMC9568611; doi:10.1038/s41467-022-33757-0)
Supplement: Supplementary file 3 — Description of Additional Supplementary Files [file 41467_2022_33757_MOESM3_ESM.pdf]

Description of Additional Supplementary Files

File Name: Supplementary Data 1
Description: Crystal data structure cif file for Pd4Sn model

File Name: Supplementary Data 2
Description: Crystal data structure cif file for PdO@Pd4Sn model

File Name: Supplementary Data 3
Description: Crystal data structure cif file for PdO(101) model
